# Health policies in Brazil (2016-2022): neoliberalism, pandemic, and threats to the Brazilian Unified National Health System

**DOI:** 10.1590/0102-311XEN228525

**Published:** 2026-07-31

**Authors:** Cristiani Vieira Machado, Luciana Dias de Lima

**Affiliations:** 1 Escola Nacional de Saúde Pública Sergio Arouca, Fundação Oswaldo Cruz, Fiocruz, Rio de Janeiro, Brasil.

**Keywords:** Health Policy, COVID-19 Pandemic, Health Systems, Unified Health System, Latin America, Política de Salud, Pandemia de COVID-19, Sistemas de Salud, Sistema Único de Salud, América Latina

## Abstract

The article analyzes health policies in Brazil from 2016 to 2022, a period marked by a shift in national politics and the COVID-19 pandemic. The study examines federal health policy management under the administrations of Michel Temer (2016-2018) and Jair Bolsonaro (2019-2022), considering the context, the political process, and policy content, as well as the different phases of the pandemic response. Drawing on historical institutionalism and the concept of critical juncture, the analysis explores continuities, changes, and disputes surrounding the Brazilian Unified National Health System (SUS). The study drew on document research, news articles, and the academic literature. The results indicate that the period was a critical juncture, with immediate and severe effects on policy implementation and the pandemic response. There were constraints on sectoral funding, setbacks in specific policies, institutional weakening, and forms of resistance within the state and society. During the period studied, no radical changes were observed in the direction of the Brazilian health system, due to structural factors, the institutional density of the SUS, competing policy agendas, the interaction of coalitions of actors with varied interests, or even the period’s short duration. However, fully understanding the impact of this critical juncture, which is still recent, requires assessing its medium- and long-term repercussions at the global, regional, and national levels.

## Abstract (es)

Este artículo analiza las políticas de salud en Brasil entre 2016 y 2022, un período marcado por una inflexión política nacional y la pandemia de la COVID-19. Se examina la gestión federal de la política de salud en los Gobiernos Michel Temer (2016-2018) y Jair Bolsonaro (2019-2022) según el contexto, el proceso político y el contenido de las políticas, así como los diferentes momentos de la respuesta a la pandemia. Con base en el institucionalismo histórico y en el concepto de coyuntura crítica, el análisis explora continuidades, cambios y disputas en torno al Sistema Único de Salud (SUS). Este estudio se fundamentó en investigaciones documentales, artículos periodísticos y en la literatura sobre el tema. Los resultados revelan que el período se configuró como una coyuntura crítica, con efectos inmediatos y graves en la gestión de las políticas y la respuesta a la pandemia. Hubo limitaciones en el financiamiento sectorial, retrocesos en políticas específicas, debilitamiento institucional y formas de resistencia dentro del Estado y la sociedad. Durante el período estudiado, no se observaron cambios radicales en la dirección del sistema de salud brasileño a causa de factores estructurales, de densidad institucional del SUS, de la disputa entre proyectos, de la interacción de coaliciones de actores con intereses variados o incluso su corta duración. Sin embargo, comprender el impacto de esta coyuntura crítica que aún es reciente requiere investigar sus repercusiones a mediano y largo plazo, a nivel global, regional y nacional.

## Introduction

The Brazilian health reform, in the context of the redemocratization of the 1980s, culminated in the creation of a public and universal Brazilian Unified National Health System (SUS, acronym in Portuguese). In the following decades, the implementation of the SUS, along with the expansion of social rights, led to the expansion of public services nationwide and improvements in health indicators. Key aspects of this process include consolidating primary health care (PHC), especially through the Family Health Strategy (FHS); expanding access to health services, supplies, and technologies of varying complexity; and institutionalizing comprehensive policies, such as the organization of health care networks. This path, however, was marked by persistent contradictions, such as health inequalities, insufficient public funding, and a strong private sector that expanded and diversified during the period ^1^.

Despite these limitations, Brazilian democracy sustained important institutional advances in health, including increased social participation and the affirmation of the right to health as a duty of the State for more than three decades. This process, however, suffered a turning point following the political crisis of 2014-2015. The impeachment of President Dilma Rousseff in 2016 marked a rupture in the prevailing political-institutional arrangement. It paved the way for the rise of neoliberal, conservative ^2^ and authoritarian ^3^ federal governments. This inflection, often characterized as a “turn to the right” ^4^, imposed constraints on democracy ^5^ and had a transversal impact on public policies ^3^, including health ^6^. 

Between 2016 and 2022, a period corresponding to the governments of Presidents Michel Temer and Jair Messias Bolsonaro, health policies were impacted by this new political-institutional context and by the COVID-19 pandemic, which hit Brazil from 2020 onwards. The magnitude of the multidimensional crisis related to the health emergency, which led to millions of deaths worldwide − more than 700,000 of them in Brazil − in addition to the worsening of economic, social, and health indicators, sparked international and national debates regarding a possible transformation of the pattern of state intervention in public policies ^7^ ^,^ ^8^.

National studies that have analyzed key aspects of this period − the effects of fiscal austerity on the financing of the SUS ^9^ ^,^ ^10^, the dismantling of strategic policies ^6^, the federal management of the pandemic ^11^ − contribute to understanding the threats and disputes surrounding the Brazilian health system. 

This article engages with this literature but proposes a specific analytical focus: examining the trajectory of national health policies throughout the Temer and Bolsonaro governments, considering the political inflection of 2016 and the COVID-19 pandemic as components of a complex period in which critical events and the interaction of diverse political forces could alter the course of the SUS. In this sense, by drawing on historical institutionalism and the concept of critical conjuncture, the study seeks to understand to what extent this period opened possibilities for radical, potentially lasting institutional changes, for the enhancement or retraction of the SUS, or, conversely, reinforced previous trajectories. 

To this end, the article was organized as follows. Initially, health policies in the two governments are described and interpreted separately, with attention to their contexts, the political processes, and their contents. Following this, the meaning and possible institutional repercussions of the changes during this period are discussed. 

The analysis was guided by the following questions: to what extent did the period constitute a critical juncture for health policies? How did the political shift of 2016 impact national health policies and the SUS? Did the political inflection and the COVID-19 pandemic produce relevant institutional transformations, or did elements of continuity prevail? 

## Methodology 

The study analyzed national health policies in Brazil from 2016 to 2022. The period began with the opening of the impeachment process, the removal of Dilma Rousseff, and the assumption of the Presidency by Michel Temer (May 2016). It ended with the end of Jair Bolsonaro’s government (December 2022).

The trajectory of health policies was characterized using the “policy triangle” ^12^, considering the context, the political process, and the policy content in the Temer (May 2016 to December 2018) and Bolsonaro (January 2019 to December 2022) governments. 

The study drew on historical institutionalism, emphasizing the role of the State, organizations, formal rules, and informal procedures that structure conduct, as well as the context, trajectory, and dynamics of policy continuities and changes ^13^. 

Given the critical nature of events during the period − a presidential impeachment, the assumption of power by right-wing leaders, and the COVID-19 pandemic − contributions from the literature on critical junctures were considered. This refers to moments of relative structural indeterminacy and fluidity in which different options for institutional innovation present themselves. In such moments, even under the constraints of the previous trajectory and a range of options, political actors would have more choices, which can affect the course of policy. In other words, in a context of uncertainties, political action is decisive, as actors can make decisions that would be improbable in scenarios of stability ^14^. This analytical framework helped to examine whether the inflections in the political-institutional and health contexts had driven transformations in the State’s role in health and in the prospects for consolidating the SUS, and in what sense these transformations had occurred. 

The main research technique was document analysis, encompassing laws and regulations, institutional websites, official documents, and reports. Box 1 summarizes the documents selected based on direct monitoring of health policy and literature, given their impact on the rules of strategic health policies and the position of the actors involved in such policies. The document analysis was complemented by a bibliographic review, journalistic articles, and consultations of testimonies and interviews with actors active during the period, available in open access.

**Box 1:** Main documents analyzed in the study.

TYPE	AUTHORSHIP	IDENTIFICATION	DATE	THEME	DESCRIPTION
Constitutional Amendment	House of Representatives/ Federal Senate	*Constitutional Amendment n. 86*	17/Mar/2015	Federal funding	- Makes the execution of the parliamentary amendment schedule mandatory, limited to 1.2% of the Executive’s budget project, half allocated to health (“mandatory nature of budgetary amendments”) https://www.planalto.gov.br/ccivil_03/constituicao/emendas/emc/emc86.htm
Party document	Ulysses Guimarães Foundation /PMDB	*A Bridge to the Future*	29/Oct/2015	Proposal for national reforms	- Emphasizes economic reforms guided by fiscal adjustment, spending restraint, incentives for the private sector, and pension reform https://www.mdb-rs.org.br/fl_adm/uploads/documentos/Uma_ponte_para_o_futuro.pdf
Constitutional Amendment	House of Representatives/ Federal Senate	*Constitutional Amendment n. 95*	15/Dec/2016	Federal funding	- Imposes limits on public spending, affecting the health budget (“spending cap”) https://www.planalto.gov.br/ccivil_03/constituicao/emendas/emc/emc95.htm
Ordinance	Brazilian Ministry of Health	*Ordinance n. 1,482*	25/Oct/2016	Mental health and drugs	- Includes therapeutic communities in the National Registry of Health Establishments, so that they can receive resources from the Brazilian Unified National Health System (SUS, acronym in Portuguese) https://bvsms.saude.gov.br/bvs/saudelegis/sas/2016/prt1482_25_10_2016.html
Ordinance	Brazilian Ministry of Health	*Ordinance n. 2,436*	21/Sep/2017	Primary care	- New National Primary Care Policy - Changes rules regarding the composition of teams; a minimum number of community health workers (CHW) per team is no longer required https://bvsms.saude.gov.br/bvs/saudelegis/gm/2017/prt2436_22_09_2017.html
Ordinance	Brazilian Ministry of Health	*Ordinance n. 3,588*	21/Dec/2017	Mental health and drugs	- Changes rules relating to the Psychosocial Care Network, giving greater prominence to specialized psychiatric hospitals https://bvsms.saude.gov.br/bvs/saudelegis/gm/2017/prt3588_22_12_2017.html
Ordinance	Brazilian Ministry of Health	*Ordinance n. 3,992*	28/Dec/2017	Federal funding	- Reduces blocks of budgetary transfers from the Brazilian Ministry of Health to two expense groups - operating costs and investment - replacing the linkage to health programs https://bvsms.saude.gov.br/bvs/saudelegis/gm/2017/prt3992_28_12_2017.html
Technical Note	Brazilian Ministry of Health	*Technical Note n. 11*	4/Feb/2019	Mental health and drugs	- Changes the National Mental Health Policy and the Guidelines of the National Drug Policy; compiles resolutions from the Tripartite Interagency Committee (CIT, acronym in Portuguese) and ordinances from the Ministry published between 2017 and 2018 that alter these policies https://cetadobserva.ufba.br/sites/cetadobserva.ufba.br/files/nota_tecnica_-_esclarecimentos_sobre_as_mudancas_da_politica_de_saude_mental.pdf
Decree	Presidency of the Republic	*Decree n. 9,759*	11/Apr/2019	Collegiate bodies	- Extinguishes and establishes guidelines, rules, and limitations for collegiate bodies of the federal public administration https://www.planalto.gov.br/ccivil_03/_ato2019-2022/2019/decreto/d9759.htm
Decree	Presidency of the Republic	*Decree n. 9,761*	11/Apr/2019	Mental health and drugs	- Approves the National Drug Policy https://www.planalto.gov.br/ccivil_03/_ato2019-2022/2019/decreto/d9761.htm
Ordinance	Brazilian Ministry of Health	*Ordinance n. 188*	3/Feb/2020	COVID-19	- Declares a Public Health Emergency of National Importance due to COVID-19 https://bvsms.saude.gov.br/bvs/saudelegis/gm/2020/prt0188_04_02_2020.html
Law	Presidency of the Republic	*Law n. 13,979*	6/Feb/2020	COVID-19	- Provides for measures to address the Public Health Emergency of International Importance https://www.planalto.gov.br/ccivil_03/_ato2019-2022/2020/lei/l13979.htm
Ordinance	Brazilian Ministry of Health	*Ordinance GM/MS n. 2,282*	27/Aug/2020	Women’s Health/Sexual and Reproductive Rights	- Amends procedures for authorizing the interruption of pregnancy in the SUS, in cases permitted by law - Physicians, healthcare professionals, or those responsible for the establishment must report cases of suspected or confirmed sexual violence to the police - The medical team must offer the pregnant woman the opportunity to view the fetus or embryo via ultrasound https://www.in.gov.br/en/web/dou/-/portaria-n-2.282-de-27-de-agosto-de-2020-274644814
Ordinance	Brazilian Ministry of Health	*Ordinance GM/MS n. 2,561*	23/Sep/2020	Women’s health/Sexual and reproductive rights	- Replaces *Ordinance 2,282/2020*, after receiving criticism and contributions - Maintains the guidance for reporting sexual violence to the police https://bvsms.saude.gov.br/bvs/saudelegis/gm/2020/prt2561_24_09_2020.html
Report	Federal Senate	*Final Report of the Pandemic Parliamentary Commission of Inquiry.*	26/Oct/2021	COVID-19	- Points out errors and omissions by the Federal Government in addressing the pandemic and recommends the indictment of President Bolsonaro and other government officials for crimes of responsibility, as well as proposing new legislative measures https://legis.senado.leg.br/atividade/comissoes/comissao/2441/mna/relatorios
Ordinance	Brazilian Ministry of Health	*Ordinance GM/MS n. 715*	4/Apr/2022	Maternal and child care	- Establishes the Maternal and Child Care Network, which replaces Stork Network (*Rede Cegonha*) https://bvsms.saude.gov.br/bvs/saudelegis/gm/2022/prt0715_06_04_2022.html
Ordinance	Brazilian Ministry of Health	*Ordinance GM/MS n. 2,228*	1/Jul/2022	Maternal and child care	- Addresses the accreditation and financing of the Maternal and Child Care Network https://bvsms.saude.gov.br/bvs/saudelegis/gm/2022/prt2228_01_07_2022.html
Ordinance	Brazilian Ministry of Health	*Ordinance GM/MS n. 4,809*	30/Dec/2022	Collaboration with society for the Economic-Industrial Complex of Health	- Establishes the Permanent Forum for Articulation with Civil Society - Criticized for being published in the final days of the government, without sufficient debate and without the inclusion of the National Health Council https://bvsms.saude.gov.br/bvs/saudelegis/gm/2022/prt4809_30_12_2022.html
Court Decision	Supreme Federal Court (STF, acronym in Portuguese)	STF Judgment - *Criminal Prosecution (AP) 2,668*	11/Sep/2025	Attempted *coup d’état*	- Judgment of the STF’s decision in AP 2,668, which, by majority vote, convicted eight defendants in the indictment related to the attempted *coup d’état*, including former President Jair Bolsonaro and former high-rank officials of his government https://noticias-stf-wp-prd.s3.sa-east-1.amazonaws.com/wp-content/uploads/wpallimport/uploads/2025/10/22075642/downloadPeca.pdf
International agreement	World Health Organization	*WHO Pandemic Agreement*	20/May/2025	Pandemics	- Multilateral agreement approved at the 78th World Health Assembly in 2025, after three years of negotiation, with recommendations aimed at prevention, preparedness, and response to pandemics, to be followed by member states, aiming for greater global security and equity https://apps.who.int/gb/ebwha/pdf_files/WHA78/A78_R1-en.pdf

Source: prepared by the authors based on various sources, as indicated in the links.

## Temer government: health in the face of the neoliberal agenda of the “bridge to the future”

The rise of the Vice-president Michel Temer to the Presidency of the Republic in 2016, after the impeachment of Dilma Rousseff, redefined the orientation of national policies. This shift had been anticipated by the Brazilian Democratic Movement Party (PMDB, acronym in Portuguese) in the document *Uma Ponte para o Futuro* ^15^ (*A Bridge to the Future*), published in October 2015, eight months before the Dilma’s removal (Box 1). The document proposed a reconfiguration of the State’s role, centered on reducing public spending, making social rights more flexible, and valuing the private sector. These guidelines expressed a return to the neoliberal agenda, partially contained in the previous decade, and translated into restrictions on national development and social rights ^6^. The text incorporated proposals from the presidential candidate of the Brazilian Social Democracy Party (PSDB, acronym in Portuguese), Aécio Neves, who was defeated at the elections in 2014 ^16^.

A legitimacy crisis and the rapid implementation of this agenda marked the beginning of the Temer government. The ministerial configuration and the profile of the ministers expressed the conservative orientation of the coalition that supported him. The new cabinet was formed exclusively by white men, many of them with ties to the private sector and the National Congress. Strategic ministries, such as Culture, Science and Technology, and Women, Racial Equality and Human Rights, were shut down or merged, which led to institutional weakening of areas focused on knowledge production, diversity, and citizenship. 

The government’s economic policy was based on regressive structural reforms that prioritized restoring market confidence and fiscal adjustment. The 2017 labor reform altered more than 100 provisions of the Consolidation of Labor Laws (CLT, acronym in Portuguese), expanding outsourcing opportunities, making contracts more flexible, and reducing social protection for workers ^17^. Discussions advanced on pension reform, which included raising the minimum retirement age and restrictive rules for accessing benefits, although it was not fully approved during the term. Despite the measures, in 2018, gross domestic product (GDP) grew by only 1.1% and overall economic productivity fell by 1.1% compared to 2017 ^18^. Between 2016 and 2018, rates of informal employment and unemployment increased ^19^. 

*Constitutional Amendment n. 95/2016* ^20^ represented the main landmark of the Temer government’s fiscal austerity ^21^. By freezing the Union’s primary expenditures for 20 years - the “spending cap” - the amendment subjected social investment, including in health and education, to permanent fiscal adjustment, compromising its financial viability ^22^. Furthermore, the implementation of the “mandatory budget”, established by *Constitutional Amendment n. 86/2015* ^23^, exacerbated the financial constraints, by making the execution of individual parliamentary amendments compulsory up to 1.2% of the Union’s Net Current Revenue, linking half of them to health, and including them in the sector’s constitutional minimum. The result was the strengthening of parliamentary control over resource allocation, alongside institutional fragmentation in the definition of budgetary priorities ^24^.

In this context, the structural underfunding of health deepened. Under fiscal austerity ^9^, from 2018 onwards, the Union kept minimum health spending frozen in real terms at the previous year’s level, without any link to the growth of federal revenues. The State’s capacity to invest, expand, and improve services was reduced ^10^, particularly affecting primary care, health surveillance, and the supply of essential medicines. 

Federal transfers to states and municipalities fell short of the SUS’s needs, widening regional inequalities and pressuring on municipal and state governments. In 2017, the reduction of the Brazilian Ministry of Health’s transfer blocks to only two expenditure groups − operating costs and investment - raised concerns regarding the federal coordination capacity, given the gains in autonomy of subnational entities ^25^. Simultaneously, parliamentary amendments gained relevance in the allocation of federal resources among municipalities ^26^.

From a political perspective, the health ministers of the period expressed, in different ways, this neoliberal shift, with discontinuities and threats to the institutional framework of the SUS. The appointment of Ricardo Barros as Minister of Health in May 2016, in recognition of his party’s support for the impeachment of President Rousseff, symbolized the convergence of the private sector’s interests with the direction of public policy. An engineer with a parliamentary background, supported by healthcare companies, Barros publicly declared at the beginning of his administration that constitutional rights would not be sustainable given budgetary constraints and advocated for reviewing the scope of the SUS ^27^.

His main initiative was the proposal for “popular health plans”. To this end, a working group was formed with representatives from the Ministry of Health, the Brazilian National Regulatory Agency for Private Health Insurance and Plans, and the National Confederation of General Insurance, Pension, Life Insurance, Health Insurance, and Prize-Linked Savings Companies. The proposal envisioned plans with reduced coverage and low cost, aimed at the low-income population. Under the argument of “expanding access”, it implied deregulation of the supplementary sector and expansion of the insurance market, but was rejected following technical analysis and public participation ^28^. 

Ricardo Barros’s administration was also marked by the publication of the new National Primary Health Care Policy, which generated controversy by making the organization of primary care, the composition of teams, and professionals’ work hours more flexible ^29^. Mental health policies suffered setbacks due to changes in the rules of the psychosocial care network, which reinforced therapeutic communities and psychiatric hospitals ^30^.

The brief administration of Gilberto Occhi (April to December 2018), Temer’s last Minister of Health, with a background in public management and a career at the Federal Savings Bank, maintained the focus on administrative rationalization and budgetary control, without presenting structural proposals for strengthening the SUS. 

The Brazilian National Health Council (CNS, acronym in Portuguese) resisted threats to the SUS during the Temer government ^31^ through dialogue and political confrontation, including through legal means. The CNS challenged the fiscal adjustment associated with *Constitutional Amendment 95/2016* ^20^ and defended the public and universal nature of the SUS, even in the face of government boycotts of its deliberative role.

In summary, the Temer government promoted a reorientation of social policies in Brazil, based on fiscal restraint, a reduction in the State’s role, and the expansion of market mechanisms, thereby harming the trajectory of health policy expansion from previous decades. The impact of *Constitutional Amendment 95/2016* on federal funding, coupled with regressive reforms and the deregulation of strategic sectors, weakened the state’s and the SUS’s capacity to guarantee the right to health. This set of measures prepared the ground for greater setbacks in the subsequent government.

## Bolsonaro government: discontinuities, pandemic, and denialism

Jair Bolsonaro, elected president of Brazil in October 2018, is a far-right politician, a retired Army captain since 1988, and a federal representative for seven terms, identified with defending the interests of the military and police. While he shares common agendas with other conservative politicians, a distinguishing feature is his defense of the military dictatorship, as explicitly stated in media interviews and his parliamentary work ^4^.

Political and institutional factors favored Bolsonaro’s victory in a critical context. The unfolding of the Federal Police’s Operation Car Wash, including the imprisonment of Luiz Inácio Lula da Silva and the impeachment of President Rousseff, supported by state actors (Attorney General’s Office, Supreme Federal Court, National Congress), political and economic elites, and the media, contributed to the weakening of democracy, the conservative shift, and the the rise of far-right parties and politicians ^4^ ^,^ ^5^.

In addition to political factors, two changes in electoral rules influenced the election of Bolsonaro and parliamentarians from his support base: the short duration of the 2018 election campaign and the reduction in the deadline for changing party affiliation before the election ^4^. The attack suffered by Bolsonaro during the campaign and the selection of Fernando Haddad as the Workers’ Party (PT, acronym in Portuguese) presidential candidate to replace Lula, who was imprisoned and prevented from running, also had repercussions in the contest. Finally, the spread of false information on social media contributed to the growth of the victorious candidate ^4^. 

Bolsonaro won the 2018 elections in the second round with 55.13% of the valid votes, against Haddad’s 44.87%, having received more votes among men, while Haddad predominated among women. Except for the Northeast, Bolsonaro had the majority of votes in all regions, with a significant vote share in the largest states of the Federation: São Paulo, Minas Gerais, and Rio de Janeiro ^4^. 

The Bolsonaro government needs to be understood in two phases. The first phase corresponds to the government’s first year (2019), when a public policy reform agenda with a neoliberal, conservative, and authoritarian orientation was adopted. The second phase is marked by the response to the COVID-19 pandemic, which prompted a reorientation of strategies. However, elements of the ideological orientation, governing style, and agenda adopted in the first phase persisted. 

General characteristics of the government include: anti-political rhetoric and authoritarian populism, expressed in the way it dealt with institutions and social actors; critical discourse towards the State and bureaucracy, associated with austerity measures and restrictions on public spending; ultraliberalism, as a positive gesture to the markets; and the prominence of conservative leaders and military personnel in high-level government positions to please their electoral base ^3^. 

The number of ministries was reduced from 29 to 22 to decrease expenses and bureaucracy. The president prioritized personal loyalty and alignment with neoliberal and conservative ideas in his cabinet appointments. One of the most remarkable ministries was the ultraliberal economist Paulo Guedes in the super-ministry of Economy, wich was formed by merging four previous ministers strongly linked to the government’s orientation, who would promote setbacks in policies within ministries such as Environment, Education, Foreign Affairs, and the Ministry of Women, Family and Human Rights; and the military, whose participation in the government would increase ^3^ ^,^ ^32^. Furthermore, the president’s media presence and the interference of his three sons − a senator, a federal representative, and a city councilor − in government affairs should be highlighted. 

The liberal, conservative, and authoritarian orientation of the Bolsonaro government manifested itself in institutional reforms and public policies. There was limited emphasis on intergovernmental coordination, dialogue with communities of experts across fields, and restrictions on social participation in policy-making, including constraints on the operation of management boards (Box 1) 

Economic policy deepened changes initiated during the Temer administration, characterized by: the adoption of fiscal measures to meet the limits imposed by *Constitutional Amendment 95/2016* (pension reform, budget cuts, and decoupling of spending); the implementation of labor reform, which reduced workers’ rights; privatizations of companies and changes in state-owned enterprises. Other policies that showed setbacks were: foreign policy, with an erosin of Brazil’s international standing; environmental policy, due to weakened regulation; science and technology policy, due to low investment; and education policy, due to budget restrictions, incentives for the private sector, and conservatism. Policies related to gender, human rights, and sexual and reproductive rights have been affected, impacting strategies aimed at women, the LGBTQIA+ population, and the promotion of racial equality ^3^. The term “dismantling” has been used to highlight the deconstruction and magnitude of the setbacks in social policies, which have impacted health ^6^.

In health policy, the first year of the government (2019) was marked by controversial changes at the Ministry of Health and in strategic policies, influenced by the federal government’s positions. The first Minister of Health of the period, Luiz Henrique Mandetta, was a federal representative for the State of Mato Grosso do Sul, from a conservative party, with experience as a military physician, municipal health secretary, and in the private health sector. 

The first controversy concerned the interruption of the *Mais Médicos* Program, a landmark of Dilma Rousseff’s government that aimed to provide physicians to areas of social vulnerability, as announced by Bolsonaro during his presidential campaign. Medical entities had criticized this program for incorporating foreign professionals − especially Cubans − without validating their diplomas. At the end of 2018, after the elections, the Cuban government suspended the agreement with Brazil, mediated by the Pan American Health Organization (PAHO), withdrawing most of the Cuban physicians from the country. 

Under Mandetta’s administration, the Bolsonaro government launched the *Médicos pelo Brasil* Program, with similar objectives, but requiring national validation of diplomas obtained abroad. In practice, implementation of the new program was delayed, and both initiatives coexisted. Other changes in PHC policies included alterations to financing rules − replacing per capita and per-team transfers with transfers conditioned on individual registration − and increased flexibility in the composition of Family Health teams. In summary, changes in PHC policy reflected an alignment with the interests of medical professional associations, as well as a managerialist and pro-market orientation.

The second controversial measure was the elimination of the Department of Sexually Transmitted Infections, AIDS, and Viral Hepatitis within the Brazilian Ministry of Health, which was replaced by the Department of Chronic Diseases and Sexually Transmitted Infections. This change was criticized by public health experts and social movements fighting against AIDS, as it subordinated the coordination of this policy to the structure responsible for chronic diseases. Concerns were related to the government’s conservative agenda, unfavorable to the demands and rights of the LGBTQIA+ population. 

In mental health policies, measures adopted during the Temer administration that represented setbacks were reiterated, such as the strengthening of private therapeutic communities and psychiatric hospitals ^30^. In the area of women’s health and sexual and reproductive rights, changes in the maternal and child care network and rules that would hinder access to pregnancy termination in cases provided for by law stand out (Box 1) ^33^. Tobacco control policies, which require intergovernmental coordination and engagement with society, were hampered by low priority and weakening of collegiate bodies ^34^, risking the achievement of their goals ^35^.

Health policies under the Bolsonaro government, however, would be marked by the management of the COVID-19 pandemic, which hit the country in 2020, resulting in almost 700,000 deaths by the end of 2022. The limitations in addressing the health crisis extended beyond the health sector. They can be attributed largely to the leadership of the President and his inner circle, characterized by authoritarianism and denialism. Authoritarianism was expressed in the centralization of decision-making and the imposition of his ideas, to the detriment of dialogue with other governments, health authorities, scientists, and social groups. Denialism manifested itself in the disregard for the severity of the health emergency, the denial of scientific evidence for addressing it, and the recommendation of measures without a technical-scientific basis. 

The Brazilian Ministry of Health was marked by instability and institutional weakening, with four ministers during the period. Limitations in the national governance of the pandemic response were reflected in the underutilization of SUS bodies for intergovernmental coordination (such as the tripartite health commission) and social participation (health councils), as well as in the government’s limited dialogue with experts and diverse social groups. 

Despite these general characteristics, distinct moments can be identified in Brazil’s response to the COVID-19 pandemic (Box 2). 

**Box 2:** Moments in the fight against the COVID-19 pandemic in Brazil, 2020-2022.

ATTEMPTS AT COORDINATION BY THE MINISTRY OF HEALTH (JAN-APR 2020)	POLITICAL POLARIZATION: “HEALTH VS. ECONOMY” (APR-DEC 2020)	SLOW VACCINATION, HIGH MORTALITY, PARLIAMENTARY INVESTIGATION (JAN-JUN 2021)	EFFECTIVENESS OF VACCINATION VS. DENIALISM (JUN/2021-DEC/2022)
Limits of federal actions in border closures, economic and social measures Physical distancing measures depended on state and municipality decisions Initial preparation: new legislation, installation of the national Emergency Operations Center (COE, acronym in Portuguese), training of national laboratories and other Latin American countries for diagnosis Coordination efforts and daily reports from the Brazilian Ministry of Health Emphasis: hospital care Difficulties in importing supplies and equipment Conflicts between the positions of the President and the Brazilian Ministry of Health	Conflicts: President, health authorities, and experts Changes in the Brazilian Ministry of Health: two ministerial changes, alterations in leadership. Federal Government decisions not based on scientific evidence Resistance to physical distancing measures, pressure to normalize the economy Vaccines: technology transfer agreements vs. conflicts over purchases and effectiveness. Instability of national information systems Subnational initiatives (e.g., Northeast Consortium, involving governors, health authorities, partners), public organizations (Fiocruz, universities), media organizations, and social movements to provide reliable information about COVID-19 and promote other control measures	Domestic vaccine production (Oswaldo Cruz Foudation [Fiocruz] and Butantan Institute): dependent on imports of Active Pharmaceutical Ingredient (API) Gradual vaccination by the Brazilian Unified National Health System (SUS), according to vaccine availability. March 2021: changes in the Brazilian Ministry of Health Establishment of a Parliamentary Commission of Inquiry (CPI, acronym in Portuguese) into the federal government’s response (Pandemic CPI) Conflicts between government authorities and experts/scientists	Achieving high vaccination coverage in adults with the basic schedule protects against hospitalizations and deaths. Complete cycle of national vaccine production by Fiocruz Increased availability of vaccines and tests. Despite scientific evidence on vaccine effectiveness, fake news and anti-vaccine campaigns generate vaccine hesitancy and low coverage among children and lower coverage of booster doses in adults (after the basic schedule). Final Report of the Pandemic CPI points to omissions and crimes by authorities to be investigated

Source: prepared by authors.

The initial period, from January to April 2020, is characterized by the Brazilian Ministry of Health’s efforts to coordinate the response to COVID-19 amid the first cases in China and its rapid spread in Europe. The World Health Organization (WHO) declared a Public Health Emergency of International Concern on January 30. On February 3, with no cases registered in the country, Brazil declared a Public Health Emergency of National Concern and established the Public Health Emergency Operations Center. In February, the Ministry of Health passed a law that established rules for isolation, quarantine, mandatory notification, and other control measures. Through Oswaldo Cruz Foundation (Fiocruz), a federal institution of science and technology in health, national production of diagnostic tests began, and training was provided to laboratories in nine Latin American countries for processing COVID-19 tests. The Ministry of Health held daily television broadcasts providing information about the disease wolrdwide and in the country.

This initial phase is marked by the Ministry of Health’s prompt response, enabled by the SUS, the country’s tradition of health surveillance, and prior experience in responding to health emergencies ^36^. Hospital care was emphasized through guidelines, expansion of beds, and the organization of temporary field hospitals in several states. However, little emphasis was placed on the role of primary health care in the response.

The Ministry of Health’s recommendations, based on technical criteria and available scientific evidence, clashed with President Bolsonaro’s positions, which opposed physical distancing. Federal measures to close borders, restrict mobility, and promote physical distancing were limited and dependent on decisions by state and municipal governments. The conflicts culminated in the dismissal of Minister Mandetta in April 2020, followed by other officials.

The second phase, from April to December 2020, is marked by the exacerbation of the “health versus economy” polarization. The technical recommendation to maintain physical distancing measures, when vaccines were not yet available, met with resistance from economic agents and government officials. National measures for social protection, employment, and businesses were late and insufficient, shifting responsibility to states and municipalities. Conflicts between the president, researchers, and health experts intensified. The Brazilian Ministry of Health experienced periods of instability, with two ministerial changes. Mandetta’s successor, Nelson Teich, an oncologist, resigned after less than a month in office (in May 2020). 

A military officer, General Pazuello, was appointed Minister of Health, without specific training or experience in managing the SUS, aiming to improve logistics in the response to the crisis. The presence of military personnel in civilian health positions expanded by 74% between 2018 and 2020, mirroring the militarization of federal administration during that period. At the end of 2020, there were 10 military ministers, almost half of the total ^32^. 

To avoid conflict with the president, the Brazilian Ministry of Health did not emphasize measures recommended by experts, such as physical distancing and mask use. Furthermore, it was negligent or endorsed positions without a scientific basis, including the utilization medications with no proven efficacy against COVID-19. There were episodes of instability in the provision of COVID-19 data, with states, scientific institutions, and the media adopting alternatives to consolidate information about the disease. Another controversy was the federal government’s reluctance to sign purchase agreements with companies producing COVID-19 vaccines, which were still under development.

On the other hand, negotiations regarding technology transfer for national vaccine production advanced during this period. Fiocruz, a federal institution, led one of these processes, with the Brazilian Ministry of Health’s approval, to produce the vaccine using AstraZeneca-Oxford (United Kingdom) technology. The Butantan Institute, in the State of São Paulo, signed an agreement with the Chinese company Sinovac to produce the CoronaVac vaccine. 

The third phase, from January to June 2021, was characterized by the beginning of vaccination in the country, with vaccines produced nationally by Fiocruz and Butantan Institute. The pace of vaccine availability was hampered by reliance on imported Active Pharmaceutical Ingredients (APIs), which faced delays due to insufficient supply to meet global demand. It is worth noting the robust National Immunization Program in the country and the predominance among the Brazilian population of a positive view of vaccines as an effective intervention to prevent diseases and as a social right. Even so, vaccines were the subject of federal conflicts ^37^, and President Bolsonaro’s denial of their effectiveness encouraged the dissemination of fake news, leading to misinformation and vaccine hesitancy. In March 2021, Minister Pazuello was replaced by Marcelo Queiroga, a cardiologist, the fourth Brazilian Minister of Health during the pandemic, who would remain in office until the end of the Bolsonaro administration.

Problems in the federal response to the pandemic led Brazil to experience alarming COVID-19 mortality rates and excess overall mortality. Consequently, the Senate established a Parliamentary Commission of Inquiry − the Pandemic CPI (acronym in Portuguese) − which analyzed extensive documentation and questioned authorities and experts.

In the fourth phase, from June 2021 to December 2022, the country achieved high COVID-19 vaccination coverage, especially among adults, with scientific evidence of vaccine effectiveness in reducing hospitalizations and mortality from the disease ^38^. Fiocruz began producing the API for the AstraZeneca-Oxford vaccine and increased the supply of tests. Brazil also began purchasing vaccines from international producers, especially those using mRNA technology. Rapid self-tests became available in pharmacies. Despite evidence of the effectiveness of vaccines and the ineffectiveness of certain medications in managing COVID-19, fake news spread, fueling vaccine hesitancy, especially regarding child immunization and booster doses. 

In October 2021, the Senate released the final report of the Pandemic CPI (Box 1), recommending the indictment of more than 60 people, including President Bolsonaro, two Ministers of Health − Pazuello and Queiroga − other ministers, officials from the Brazilian Ministry of Health, three of the president’s sons, presidential advisors, parliamentarians, physicians, bloggers, businesspeople, among others. The president was indicted for ten crimes, including crimes of responsibility and crimes against humanity. The report included proposals to criminalize fake news, improve the SUS management and emergency response, hold public officials accountable, and provide support and reparations to COVID-19 victims. However, the Attorney General of the Republic archived the document.

At the end of 2022, when the Bolsonaro government ended, the country had recorded almost 700,000 deaths from COVID-19, many of which could have been avoided. There was significant excess mortality, possibly associated with other causes as well. In addition to morbidity and mortality from COVID-19, other health indicators worsened − in part because people avoided or had trouble accessing health care services during the critical stages of the pandemic − and vaccine hesitancy increased in the country. The economic and social repercussions were drastic, affecting millions of families.

Box 3 summarizes the characteristics of the Temer and Bolsonaro governments, considering elements of context, political process, and the content of health policies.

**Box 3:** Context, political process, and content of the national health policy from 2016 to 2022, according to the governments.

DIMENSION	TEMER GOVERNMENT (MAY/2016-DEC/2018)	BOLSONARO GOVERNMENT (JAN/2019-DEC/2022)
Context	- Dilma Rousseff’s impeachment process: Vice-President Temer assumes the presidency in May/2016 on an interim basis and, in August, permanently, until the end of 2018 - Neoliberal economic policies, with an emphasis on austerity and spending cuts, guided by the “*A Bridge to the Future*” program - Configuration of ministries and profile of ministers: merger or extinction of ministries, predominance of white men with a conservative profile - *Constitutional Amendment 95/2016* - spending cap. - Proposals for labor and social security reforms, with a reduction in workers’ and social rights - Imprisonment of Lula, who is prevented from running in the 2018 elections - 2018 campaign: changes in electoral rules, attack on Bolsonaro - Elections: Bolsonaro’s victory over Fernando Haddad (in the second round)	- Ministerial reform: reduction in the number of ministries through the elimination or merging of portfolios; creation of the super-ministry of economy (headed by Paulo Guedes) - Ministers aligned with a neoliberal and conservative agenda, and military personnel increased in strategic areas - Militarization in the occupation of civilian positions in the federal administration - Ultraliberal economic policies and measures to favor markets - Conservatism leads to setbacks in pro-diversity and equity policies - Setbacks in foreign, environmental, education, and other policies - COVID-19 pandemic severely impacts the country between 2020 and 2022: the federal response is marked by insufficient policy coordination, denialism, authoritarianism, and contradictions - Establishment of the Pandemic Parliamentary Commission of Inquiry (CPI, acronym in Portuguese) in 2021
Political process in health	Ministerial tenures: Ricardo Barros (May/2016-Apr/2018): - Civil engineer and businessman - Federal Representative − Progressive Party (PP − Paraná); former mayor of Maringá (Paraná State) - The Minister questions the scope of social rights and the Brazilian Unified National Health System (SUS, acronym in Portuguese) - Conservative composition in the Brazilian Ministry of Health’s secretariat Gilberto Occhi (Apr/2018-Dec/2018): - Lawyer and career employee of the Federal Savings Bank - Member of the PP; former Minister of Cities and of National Integration in the Dilma government (between 2014-2016) - Emphasis on budget management and administrative rationalization Relationships with other actors: - Synergy with the guidelines of economic authorities. - Favoring of private actors - Resistance from the Brazilian National Health Council and other sectoral actors	Ministerial tenures: Luiz Henrique Mandetta (Jan/2019-Apr/2020): - Orthopedic surgeon and rural landowner - Federal Representative - Democrats (DEM − Mato Grosso do Sul State) - Experience in public and private health management. - Mixed composition in the ministry, with a conservative profile and technical staff - Technical positions at the beginning of the COVID-19 response, generating conflicts with President Bolsonaro and the minister’s resignation Nelson Teich (Apr/2020-May/2020): - Oncologist and businessman - Resigned after less than a month in office Eduardo Pazuello (May/2020-Mar/2021): - Army General, without health training - Appointed as interim minister in May and permanent minister in September 2020. - Increasing occupation of Brazilian Ministry of Health positions by military personnel - Public alignment with the President’s guidelines in dealing with the pandemic, but authorization for national vaccine production by Oswaldo Cruz Foudation (Fiocruz, acronym in Portuguese) Marcelo Queiroga (Mar/2021-Dec/2022): - Cardiologist with management experience in the private sector - Alignment with the President - Continuity with the previous administration Relationships with other actors: - Support from national medical entities with a conservative profile - Scientific denialism by the President and other actors - Technical weakening and increasing militarization in the Brazilian Ministry of Health, with instability in pandemic management - Restrictions on collegiate bodies and social participation - Conflicts with specialists and other spheres of government - State and municipal governments adopt varied policies - Resistance from scientific institutions, health agencies (including within the Brazilian Ministry of Health, Fiocruz, and Brazilian Health Regulatory Agency), healthcare professionals, and social movements
Content of health policy	- Cost containment due to budgetary constraints (effects of EC 95/2016) - Proposal for popular healthcare plans for low-income populations: formation of a working group, but resistance and rejection - Changes in the rules for federal transfers, which are now divided into two blocks: operating costs and investments - Changes in the National Primary Care Policy (2017) with greater flexibility in team composition and the number of Community Health Agents per team - Changes in Mental Health and Drugs policies reinforce hospitals and therapeutic communities, criticized by experts for clashing with the guidelines of the Brazilian psychiatric reform and the advances of the mental health care model in the SUS	- Announcement of the discontinuation of the *Mais Médicos* program (a hallmark of the previous government) and the launch of the *Médicos pelo Brasil* program, requiring the revalidation of diplomas for physicians trained abroad. In practice, professionals hired by both programs coexisted in the country during this period - Changes in the rules for financing primary care, with a relative weakening of the Family Health Strategy - Specific policies affected by the government’s conservative agenda (e.g., women’s health, LGBTQIA+ population), by financial restrictions, and by reduced social participation - Changes in the rules relating to the maternal and child care network and authorization for the termination of pregnancy in cases permitted by law - Continuation of changes in Mental Health and Drugs policies adopted by the previous government, criticized by experts for clashing with the guidelines of the Brazilian psychiatric reform and the advances of the care model in the SUS - Management of the response to the 2020 pandemic: different moments (see Box 2) and contradictions; Tensions arise between technical strategies (e.g., decisions regarding national vaccine production) and measures lacking a scientific basis

Source: prepared by the authors, based on various bibliographic and documentary sources.

## Discussion

The period from 2016 to 2022, corresponding to the Temer and Bolsonaro governments, represented a critical juncture for health policies in Brazil, due to events at two levels. 

The first level is the political-institutional one. The political crisis and the impeachment of Dilma Rousseff − identified by many as a coup ^39^ ^,^ ^40^ − brought Temer to power. They favored adopting neoliberal reforms guided by austerity, the privileging of private interests, and a managerial approach to policy. The Bolsonaro government continued these reforms, associated with a conservative agenda and anti-democratic mechanisms, such as legal prohibitions on collegiate bodies, the increasing occupation of strategic positions by people without technical qualifications, including military personnel ^32^, disrespect for institutions, and discontinuities in public policies due to economic constraints, neoliberal ideas, or conservatism. In other words, the agendas and measures of the Temer and Bolsonaro governments clashed with the principles and conditions necessary for the consolidation of the SUS.

The second level is the health crisis, given the magnitude of the COVID-19 pandemic and its tragic repercussions in Brazil. The health emergency reached the country in an adverse political and institutional scenario, when the negative effects of the neoliberal and conservative agenda were already affecting public policies and the SUS. Although structural, institutional, and societal factors influenced Brazil’s response to the pandemic ^11^, the political and conjunctural factors, especially President Bolsonaro’s positions, were decisive in explaining the poor results observed in a country with a universal health system and solid scientific institutions. The Pandemic CPI report identified evidence of omissions or crimes committed by government officials that warrant judicial review.

In a critical juncture, on both the political, institutional, and health levels, health policies were affected by: budget cuts and instability; weakening of federal agencies; occupation of strategic posts by individuals who lacked the necessary qualification or commitment to the principles of the SUS; disrespect for mechanisms of social participation; discontinuities in policies (such as those related to primary care, mental health, women’s health, and tobacco control); denialism by government officials and decisions without a scientific basis. Therefore, the political shift from 2016 onwards had repercussions on health during the analyzed period, including institutional weakening, the growth of scientific denialism, setbacks in specific policies, worsening of health indicators, and potentially avoidable deaths in the context of the pandemic. 

On the other hand, at this critical juncture, there was resistance to dismantling institutions and policies within the State and in society. Public servants, researchers, managers, health professionals, specialist societies, participatory bodies, social movements, and some politicians mobilized to defend the SUS and the health of the population. 

While the period was a critical juncture for health, it is worth questioning whether it led to substantive institutional changes that altered the course of the Brazilian health system. Despite the frequent association between critical junctures and expectations of institutional change, the relationship between the two is not unequivocal: institutional changes can occur gradually and incrementally, not only in critical junctures ^13^ and there are critical junctures that do not lead to institutional changes ^14^. 

The critical juncture analyzed could have provoked profound institutional transformations in health in different ways. The study highlighted the complexity of this juncture and disputes between different projects for Brazil and the health system. On one hand, neoliberalism, conservatism, and authoritarianism weakened democratic institutions, state structures, and public policies, but failed to destroy them during the period analyzed. Examples of radical transformations contrary to the SUS would be: constitutional changes that altered the right to health and the rules of the system; the dismantling of state bodies and entities; the destruction of state capacities in strategic areas (regulation, health surveillance, service provision, production of supplies); the dismantling of the SUS’s collegiate bodies, federative coordination, and social participation; and the replacement of a universal model with one anchored in the expansion of private plans and insurance. 

On the other hand, the onset of the health emergency raised expectations of a shift in nation-states’ intervention towards greater responsibility in social protection and health ^7^ ^,^ ^8^. In Brazil, the political climate was unfavorable to state expansion. However, the pandemic may have slowed radical neoliberal reforms by highlighting the importance of public institutions in the immediate response to the crisis, especially scientific organizations, social protection institutions, and the SUS. Actors defending the SUS formed coalitions with expert societies, social movements, and parliamentarians of varying political orientations. For example, the National Congress authorized extraordinary budget allocations to address the emergency. The national reach of the SUS and the technical-political density of its institutions and services, supported by diverse actors and interests, were fundamental to the response, even though this was hampered by the President’s positions contrary to scientific recommendations and by the federal government’s insufficient action.

In short, the critical situation had immediate effects on health organizations and policies during the period analyzed. However, the study did not identify radical institutional transformations, in the sense of expanding or contracting the State’s role in health, structural changes in the rules of the SUS, or the destruction or strengthening of its organizations. Despite the pressures and constraints, neither health institutions nor democratic institutions were destroyed during this period. From another perspective, the expectations of State expansion and strengthening of public institutions, as a result of the crisis experience, did not materialize. 

Several factors may explain why radical transformations in Brazilian health institutions were not observed in the analyzed context. The first, of a historical-structural nature, is the unique configuration of health in the country, in which a universal health system, created more than three decades ago, coexists with a vigorous private sector that accommodates diverse interests. The second would be the institutional density of the SUS, which manifests itself in different dimensions: in the legal dimension, through the constitutional protection of the right to health and the principles of the SUS; in the organizational dimension, through the territorial capillarity of the service network; In the federative sphere, due to shared competencies, interdependence, and coordination mechanisms between levels of government; in the technical sphere, due to the accumulated knowledge and experience in public organizations and services; and in the sociopolitical sphere, due to the diversity of actors involved with the SUS, including health professionals and officials, participatory councils, epistemic communities, and social movements.

These two factors − the historical-structural configuration of health and the institutional framework of the SUS − would favor a certain “path dependence” ^13^, which would explain the relative institutional stability and its support by coalitions of actors who resist changes in formal rules. 

The third factor concerns the convergence of opposing agendas and forces in the critical juncture analyzed. On the one hand, the neoliberal, conservative, and anti-democratic agenda led to setbacks in policies and provoked resistance. On the other hand, the severity of the health emergency, by requiring immediate state intervention, may have slowed the pace of neoliberal reforms. However, the expectation that the crisis would strengthen the State and the SUS did not materialize due to the unfavorable political scenario. 

The fourth factor concerns the temporal scope of the study, given the short, recent period analyzed, which, so far, makes it impossible to identify longer-lasting or delayed effects. Regarding the political dimension, in the 2022 presidential elections, for the first time since democratization, an incumbent president − Jair Bolsonaro − was not re-elected to a second term, thereby interrupting the implementation of his government agenda. The attempted *coup d’état* following Lula’s electoral victory was thwarted, leading to the trial and conviction of several defendants. Despite the threats, Brazilian democratic institutions showed some resilience. During Lula’s third term, which began in 2023, measures adopted by the Temer and Bolsonaro governments in the health sector were reversed, requiring further study. 

This study contributes to the theoretical debate on the dynamics of public policies by highlighting that critical junctures can produce relevant political and health effects without necessarily resulting in radical structural or institutional changes, especially in contexts marked by high institutional density and disputes between antagonistic projects. However, the repercussions of a critical juncture depend on its duration and on the interactions among political agents and coalitions. 

The study’s limitations include the recent nature of the events and the lack of interviews with actors involved in the policies, partially mitigated by the diversity of documentary sources and by consultation of statements from government officials, managers, and experts available on official websites, in news articles, and in videos. Despite mentions of the composition of governments, which express their conservative or authoritarian orientation − for example, the cabinet formed by white men in the Temer government, the presence of the military in the Bolsonaro government − this study did not delve into the influence of the actors’ profiles and power relations on the direction of health policies. Further research should explore how the composition and diversity of the government, across different dimensions − partisan, regional, gender, ethnic-racial, and social base -, impact sectoral policies. Another limitation concerns the focus on national health policy, emphasizing the context and identification of changes that clashed with the principles of the SUS or previous achievements, as denounced by the expert community and the media. Thus, the study did not delve into specific policies nor explore international and subnational factors that influence health policies.

From a global health perspective, after the WHO declared the end of the Public Health Emergency of International Concern in 2023, COVID-19 began to be monitored as just another infectious disease in several countries, including Brazil. However, the crisis led to significant developments, such as the approval in 2025 of the Pandemic Agreement ^41^, whose implementation needs to be monitored.

In a context of global transformations impacting health, it is important to follow the medium- and long-term repercussions of events over the last decade, including the pandemic, considering global, regional and national factors. Furthermore, given the conflicting political agendas at the national level, it is fundamental to analyze continuities and changes in health policies across different contexts, including the quality of democracy and the political orientation of governments. In this research agenda, it is worth considering that gradual, incremental changes can have significant effects on public policies ^13^ and the direction of the Brazilian healthcare system. 

Finally, in addition to preparing for future emergencies, consolidating the SUS depends on addressing structural problems, such as insufficient funding, excessive reliance on imported supplies, and the character of state-market relations in healthcare. It also requires changes in the pattern of socio-economic development and the affirmation of democratic values, both of which are fundamental to building a less unequal society.

## Data Availability

The sources of information used in the study are indicated in the body of the article.
