# Supplementary material for: Health policies in Brazil (2016-2022): neoliberalism, pandemic, and threats to the Brazilian Unified National Health System
Source: Cad Saude Publica. 2026 Jul 31;42(Suppl 1):e00228525. doi: 10.1590/0102-311XEN228525 (PMC13435632; doi:10.1590/0102-311XEN228525)
Supplement: Translation

# Translation: Políticas de saúde no Brasil (2016-2022): neoliberalismo, pandemia e ameaças ao Sistema Único de Saúde

**DOI:** 10.1590/0102-311XPT228525

**Authors:** Cristiani Vieira Machado, Luciana Dias de Lima

**Affiliations:** 1 Escola Nacional de Saúde Pública Sergio Arouca, Fundação Oswaldo Cruz, Fiocruz, Rio de Janeiro, Brasil.

**Keywords:** Política de Saúde, Pandemia de COVID-19, Sistemas de Saúde, Sistema Único de Saúde, América Latina

## Abstract

O presente artigo analisa as políticas de saúde no Brasil entre 2016 e 2022, período marcado por uma inflexão política nacional e pela pandemia de COVID-19. O estudo examina a condução federal da política de saúde nos governos de Michel Temer (2016-2018) e Jair Bolsonaro (2019-2022), considerando o contexto, o processo político e o conteúdo das políticas, bem como os diferentes momentos da resposta à pandemia. Com base no institucionalismo histórico e no conceito de conjuntura crítica, a análise explora continuidades, mudanças e disputas em torno do Sistema Único de Saúde (SUS). O estudo apoiou-se em investigação documental, em matérias jornalísticas e na literatura sobre o tema. Os resultados apontam que o período se configurou como uma conjuntura crítica, com efeitos imediatos e graves na condução das políticas e na resposta à pandemia. Houve constrangimentos ao financiamento setorial, retrocessos em políticas específicas, fragilização institucional e formas de resistência no interior do Estado e na sociedade. Durante o período estudado, não se observaram mudanças radicais nos rumos do sistema de saúde brasileiro, devido a fatores estruturais, à densidade institucional do SUS, à disputa de projetos, à interação de coalizões de atores com interesses variados ou, até mesmo, à sua curta duração. No entanto, a compreensão do impacto dessa conjuntura crítica, ainda recente, exige a verificação de suas repercussões em médio e longo prazos, em âmbitos global, regional e nacional.

## Introdução

A reforma sanitária brasileira, no contexto da redemocratização dos anos 1980, culminou na criação do Sistema Único de Saúde (SUS), de caráter público e universal. Nas décadas seguintes, sua implementação, associada à expansão de direitos sociais, favoreceu o incremento de serviços públicos em todo o país, com a melhoria dos indicadores de saúde. Destacam-se, nesse processo, a consolidação da atenção primária à saúde (APS) − sobretudo por meio da Estratégia Saúde da Família (ESF) −, a ampliação do acesso a serviços, insumos e tecnologias de diversas complexidades e a institucionalização de políticas pautadas pela integralidade, como a organização de redes de atenção à saúde. Esse percurso, contudo, foi marcado por contradições persistentes, como desigualdades sociais, financiamento público insuficiente e um forte setor privado, que se expandiu e se diversificou no período ^1^.

Apesar desses limites, durante mais de três décadas, a democracia brasileira sustentou avanços institucionais importantes nesse setor, incluindo o aumento da participação social e a afirmação do direito à saúde como dever do Estado. Esse processo, entretanto, sofreu uma inflexão a partir da crise política de 2014-2015. O impedimento da presidenta Dilma Rousseff em 2016 marcou uma ruptura no arranjo político-institucional vigente e abriu caminho para a ascensão de governos federais de orientação neoliberal e conservadora ^2^ e, posteriormente, autoritária ^3^. Essa inflexão, frequentemente caracterizada como uma “virada à direita” ^4^, impôs constrangimentos à democracia ^5^ e repercutiu de forma transversal nas políticas públicas ^3^, inclusive na saúde ^6^. 

Entre 2016 e 2022, período correspondente aos governos dos presidentes Michel Temer e Jair Messias Bolsonaro, as políticas de saúde foram impactadas por esse novo contexto político-institucional e pela pandemia de COVID-19, que atingiu o Brasil a partir de 2020. A magnitude da crise multidimensional relacionada à emergência sanitária, que levou a milhões de mortes no mundo − mais de 700 mil no Brasil −, além da piora em indicadores econômicos, sociais e sanitários, suscitou debates internacionais e nacionais quanto a uma possível transformação do padrão da atuação do Estado nas políticas públicas ^7^ ^,^ ^8^.

Estudos nacionais que analisaram aspectos centrais desse período − os efeitos da austeridade fiscal sobre o financiamento do SUS ^9^ ^,^ ^10^, o desmonte de políticas estratégicas ^6^ e a condução federal da pandemia ^11^ - contribuem para a compreensão das ameaças e disputas em torno do sistema de saúde brasileiro. 

O presente artigo dialoga com essa literatura, mas propõe um foco analítico específico: examinar a trajetória das políticas nacionais de saúde ao longo dos governos Michel Temer e Jair Bolsonaro, considerando a inflexão política de 2016 e a pandemia de COVID-19 como componentes de um período complexo, em que acontecimentos críticos e a interação de forças políticas diversas poderiam alterar os rumos do SUS. Nesse sentido, ao adotar contribuições do institucionalismo histórico e do conceito de conjuntura crítica, este estudo busca compreender em que medida esse período ampliou as possibilidades de mudanças institucionais radicais e potencialmente duradouras − de valorização ou de retração do SUS − ou, ao contrário, reforçou trajetórias prévias. 

Para isso, o artigo foi organizado da seguinte forma: inicialmente, a política de saúde nos dois governos é descrita e interpretada separadamente, articulando elementos do contexto, do processo político e do conteúdo das políticas; na sequência, discutem-se o significado e as possíveis repercussões institucionais das mudanças nesse período. 

A análise foi orientada pelas seguintes questões: em que medida o período em questão se configurou como uma conjuntura crítica para as políticas de saúde? De que modo a inflexão política de 2016 repercutiu nas políticas nacionais de saúde e no SUS? A inflexão política e a pandemia de COVID-19 produziram transformações institucionais relevantes ou prevaleceram elementos de continuidade? 

## Metodologia 

O estudo analisou as políticas nacionais de saúde no Brasil de 2016 a 2022. O início do período foi delimitado pela abertura do processo de impedimento, com o afastamento de Dilma Rousseff e a assunção da Presidência por Michel Temer (maio de 2016), e o final, pelo término do governo de Jair Bolsonaro (dezembro de 2022).

Para a caracterização da trajetória das políticas no período, com base no “triângulo de políticas” ^12^, foram identificados os principais elementos do contexto, do processo político, das relações entre os atores e do conteúdo das políticas de saúde nos governos Michel Temer (maio de 2016 a dezembro de 2018) e Jair Bolsonaro (janeiro de 2019 a dezembro de 2022). 

A pesquisa apoiou-se no referencial do institucionalismo histórico, no que se refere à valorização do papel do Estado, das organizações, das regras formais, dos procedimentos informais que estruturam condutas, do contexto, da trajetória e da dinâmica de continuidades e mudanças nas políticas ^13^. 

Considerando o caráter crítico de acontecimentos no período − um impedimento presidencial, a ascensão de lideranças de direita e a pandemia de COVID-19 −, adotaram-se contribuições da literatura sobre conjuntura crítica (*critical juncture*). O conceito refere-se a momentos de relativa indeterminação estrutural e fluidez, nos quais surgem diferentes opções de inovação institucional. Em tais contextos, embora sob os constrangimentos da trajetória anterior, os atores políticos detêm maior liberdade de escolha, o que pode afetar os rumos das políticas. Ou seja, em uma conjuntura de incertezas, a agência política torna-se decisiva, pois permite decisões improváveis em cenários de estabilidade ^14^. Esse referencial analítico permitiu examinar se as inflexões nos contextos político-institucional e sanitário teriam impulsionado transformações na atuação do Estado na saúde e nas perspectivas de consolidação do SUS, e em que sentido elas teriam ocorrido. 

A principal técnica de pesquisa foi a análise documental, abrangendo leis e normas, sítios institucionais, documentos e relatórios oficiais. Tais fontes, resumidas no Quadro 1, foram selecionadas a partir do acompanhamento direto da política de saúde e da literatura, considerando sua relevância quanto a mudanças nas regras de políticas estratégicas para o SUS e sua repercussão junto aos atores envolvidos nelas. A análise documental foi complementada por revisão bibliográfica, por matérias jornalísticas e por consultas a depoimentos e entrevistas de atores atuantes no período, disponíveis em acesso aberto.

**Quadro 1:** Principais documentos analisados no estudo.

TIPO	AUTORIA	IDENTIFICAÇÃO	DATA	TEMA	DESCRIÇÃO
Emenda Constitucional	Câmara dos Deputados/Senado Federal	*Emenda Constitucional nº 86*	17/Mar/2015	Financiamento federal	- Torna obrigatória a execução da programação de emendas parlamentares no limite de 1,2% do projeto orçamentário do Executivo, com matade destinada à saúde (“impositividade das emendas”) https://www.planalto.gov.br/ccivil_03/constituicao/emendas/emc/emc86.htm
Documento partidário	Fundação Ulysses Guimarães/PMDB	*Uma Ponte para o Futuro*	29/Out/2015	Proposta de reformas nacionais	- Ênfase em reformas econômicas orientadas pelo ajuste fiscal, contenção de gastos, incentivos ao setor privado e reforma da previdência https://www.mdb-rs.org.br/fl_adm/uploads/documentos/Uma_ponte_para_o_futuro.pdf
Emenda Constitucional	Câmara dos Deputados/Senado Federal	*Emenda Constitucional nº 95*	15/Dez/2016	Financiamento federal	- Impõe limites aos gastos públicos, afetando o orçamento da saúde (“teto dos gastos”) https://www.planalto.gov.br/ccivil_03/constituicao/emendas/emc/emc95.htm
Portaria	Ministério de Saúde	*Portaria nº 1.482*	25/Out/2016	Saúde mental e drogas	- Inclui as comunidades terapêuticas no Cadastro Nacional de Estabelecimentos de Saúde, de forma que possam receber recursos do Sistema Único de Saúde (SUS) https://bvsms.saude.gov.br/bvs/saudelegis/sas/2016/prt1482_25_10_2016.html
Portaria	Ministério da Saúde	*Portaria nº 2.436*	21/Set/2017	Atenção básica	- Nova Política Nacional de Atenção Básica - Altera regras relativas à composição das equipes; deixa de exigir número mínimo de agetes comunitários de saúde (ACS) por equipe https://bvsms.saude.gov.br/bvs/saudelegis/gm/2017/prt2436_22_09_2017.html
Portaria	Ministério da Saúde	*Portaria nº 3.588*	21/Dez/2017	Saúde mental e drogas	- Altera regras relativas à Rede de Atenção Psicossocial, conferindo maior destaque a hospital psiquiátrico especializado https://bvsms.saude.gov.br/bvs/saudelegis/gm/2017/prt3588_22_12_2017.html
Portaria	Ministério da Saúde	*Portaria nº 3.992*	28/Dez/2017	Financiamento federal	- Reduz blocos de transferências do Ministério da Saúde para dois grupos de despesas − custeio e investimento − , em substituição à vinculação a programas de saúde https://bvsms.saude.gov.br/bvs/saudelegis/gm/2017/prt3992_28_12_2017.html
Nota Técnica	Ministério da Saúde	*Nota Técnica nº 11*	04/Fev/2019	Saúde mental e drogas	- Mudanças na Política Nacional de Saúde Mental e nas Diretrizes da Política Nacional sobre Drogas; reúne resoluções da Comissão Intergestores Tripartite (CIT) e portarias do Ministério publicadas entre 2017 e 2018 que alteram tais políticas https://cetadobserva.ufba.br/sites/cetadobserva.ufba.br/files/nota_tecnica_-_esclarecimentos_sobre_as_mudancas_da_politica_de_saude_mental.pdf
Decreto	Presidência da República	*Decreto nº 9.759*	11/Abr/2019	Colegiados de gestão	- Extingue e estabelece diretrizes, regras e limitações para colegiados da administração pública federal https://www.planalto.gov.br/ccivil_03/_ato2019-2022/2019/decreto/d9759.htm
Decreto	Presidência da República	*Decreto nº 9.761*	11/Abr/2019	Saúde mental e drogas	- Aprova a Política Nacional de Drogas https://www.planalto.gov.br/ccivil_03/_ato2019-2022/2019/decreto/d9761.htm
Portaria	Ministério da Saúde	*Portaria nº 188*	03/Fev/2020	COVID-19	- Declara Emergência em Saúde Pública de Importância Nacional em decorrência da COVID-19 https://bvsms.saude.gov.br/bvs/saudelegis/gm/2020/prt0188_04_02_2020.html
Lei	Presidência da República	*Lei nº 13.979*	06/Fev/2020	COVID-19	- Dispõe sobre as medidas para enfrentamento da Emergência de Saúde Pública de Importância Internacional https://www.planalto.gov.br/ccivil_03/_ato2019-2022/2020/lei/l13979.htm
Portaria	Ministério da Saúde	*Portaria GM/MS nº 2.282*	27/Ago/2020	Saúde da mulher/direitos sexuais e reprodutivos	- Altera procedimentos para autorização da interrupção da gravidez no SUS, nos casos previstos em Lei - Médicos, profissionais de saúde ou responsáveis pelo estabelecimento devem comunicar à polícia casos de indícios ou confirmação de violência sexual - A equipe médica deve oferecer à gestante a visualização do feto ou embrião por ultrassonografia https://www.in.gov.br/en/web/dou/-/portaria-n-2.282-de-27-de-agosto-de-2020-274644814
Portaria	Ministério da Saúde	*Portaria GM/MS nº 2.561*	23/Set/2020	Saúde da mulher/direitos sexuais e reprodutivos	- Substitui a *Portaria nº 2.282/2020*, após recebimento de críticas e contribuições - Mantém a orientação para a comunicação de violência sexual à polícia https://bvsms.saude.gov.br/bvs/saudelegis/gm/2020/prt2561_24_09_2020.html
Relatório	Senado Federal	*Relatório Final da Comissão Parlamentar de Inquérito da Pandemia*	26/10/2021	COVID-19	- Aponta erros e omissões do Governo Federal no enfrentamento da pandemia e recomenda o indiciamento do presidente Bolsonaro e outros governantes por crimes de responsabilidade, bem como propõe novas medidas legislativas https://legis.senado.leg.br/atividade/comissoes/comissao/2441/mna/relatorios
Portaria	Ministério da Saúde	*Portaria GM/MS nº 715*	04/Abr/2022	Atenção Materna e Infantil	- Institui a Rede de Atenção Materna e Infantil que substitui a “Rede Cegonha” https://bvsms.saude.gov.br/bvs/saudelegis/gm/2022/prt0715_06_04_2022.html
Portaria	Ministério da Saúde	*Portaria GM/MS nº 2.228*	01/Jul/2022	Atenção Materna e Infantil	- Trata da habilitação e o financiamento da Rede de Atenção Materna e Infantil https://bvsms.saude.gov.br/bvs/saudelegis/gm/2022/prt2228_01_07_2022.html
Portaria	Ministério da Saúde	*Portaria GM/MS nº 4.809*	30/Dez/2022	Articulação com a sociedade para o Complexo Econômico-Industrial da Saúde	- Institui o Fórum Permanente de Articulação com a Sociedade Civil - Criticada por ter sido publicada nos últimos dias de governo, sem suficiente debate e sem a inclusão do Conselho Nacional de Saúde https://bvsms.saude.gov.br/bvs/saudelegis/gm/2022/prt4809_30_12_2022.html
Acórdão Judicial	Supremo Tribunal Federal (STF)	*Acórdão STF - Ação Penal 2.668*	11/Set/2025	Tentativa de golpe de Estado	- Acórdão da decisão do STF na AP 2.668 que condenou, por maioria de votos, oito réus da denúncia relativa à tentativa de golpe de Estado, incluindo o ex-presidente da República Jair Bolsonaro e ex-integrantes do primeiro escalão de seu governo https://noticias-stf-wp-prd.s3.sa-east-1.amazonaws.com/wp-content/uploads/wpallimport/uploads/2025/10/22075642/downloadPeca.pdf
Acordo internacional	Organização Mundial da Saúde	*Acordo sobre Pandemias da OMS* (WHO Pandemic Agreement)	20/Mai/2025	Pandemias	- Acordo multilateral aprovado na 78ª Assembleia Mundial de Saúde em 2025, após três anos de negociação, com recomendações voltadas à prevenção, preparação e resposta a pandemias, a serem seguidas pelos Estados-membros, visando maior segurança e equidade global https://apps.who.int/gb/ebwha/pdf_files/WHA78/A78_R1-en.pdf

Fonte: elaboração própria a partir de diversas fontes, conforme indicado nos links.

### Governo Temer: a saúde diante da agenda neoliberal da “ponte para o futuro”

A ascensão do então vice-presidente Michel Temer à Presidência da República, em 2016, após o impedimento de Dilma Rousseff, redefiniu a orientação das políticas nacionais. Essa guinada havia sido antecipada pelo Partido Movimento Democrático Brasileiro (PMDB), no documento *Uma Ponte para o Futuro* ^15^, publicado em outubro de 2015, oito meses antes do afastamento da presidenta. O documento propunha uma reconfiguração do papel do Estado, centrada na redução dos gastos públicos, na flexibilização de direitos sociais e na valorização do setor privado. Tais diretrizes expressaram a retomada da agenda neoliberal, parcialmente contida na década anterior, traduzindo-se em restrições ao desenvolvimento nacional e aos direitos sociais ^6^. O texto incorporava propostas do candidato à presidência do Partido da Social-Democracia Brasileira (PSDB), Aécio Neves, derrotado nas urnas em 2014 ^16^.

O início do governo Michael Temer foi marcado por uma crise de legitimidade e pela rápida implementação dessa agenda. A configuração e o perfil dos ministros expressaram a orientação conservadora da coalizão que o sustentava. O novo gabinete foi formado exclusivamente por homens brancos, muitos deles com vínculos empresariais e com o Congresso Nacional. Foram extintos ou fundidos ministérios estratégicos, como o da Cultura, da Ciência e Tecnologia, e o das Mulheres, da Igualdade Racial e dos Direitos Humanos, o que representou o enfraquecimento institucional de áreas voltadas à produção de conhecimento, à diversidade e à cidadania. 

A orientação econômica do governo baseou-se em reformas estruturais regressivas, que priorizavam a restauração da confiança do mercado e o ajuste fiscal. A reforma trabalhista de 2017 alterou mais de cem dispositivos da Consolidação das Leis do Trabalho (CLT), ampliando possibilidades de terceirização, flexibilizando contratos e reduzindo a proteção social dos trabalhadores ^17^. Avançaram as discussões sobre a reforma da previdência, que previa o aumento da idade mínima e regras restritivas de acesso a benefícios, embora não tenha sido integralmente aprovada durante o mandato. Apesar das medidas, em 2018, o produto interno bruto (PIB) cresceu apenas 1,1%, enquanto a produtividade geral da economia caiu 1,1% em relação a 2017 ^18^. Entre 2016 e 2018 ampliaram-se as taxas de informalidade laboral e de desocupação ^19^. 

A *Emenda Constitucional nº 95/2016* ^20^ (EC nº 95) representou o principal marco da austeridade fiscal do governo Temer ^21^. Ao estabelecer o congelamento das despesas primárias da União por vinte anos − o “teto de gastos” −, incluindo da saúde e da educação, a emenda submeteu o investimento social a um ajuste fiscal permanente ^22^, comprometendo sua viabilidade financeira. Ademais, a vigência do “orçamento impositivo” (EC nº 86, de 17 de março de 2015 ^23^) acentuou esse quadro ao tornar obrigatória a execução de emendas individuais em até 1,2% da Receita Corrente Líquida (RCL) da União. Como metade desses recursos deveria ser vinculada à saúde, eles passaram a ser contabilizados no piso constitucional do setor, fortalecendo o controle parlamentar sobre a alocação de recursos, com fragmentação institucional na definição das prioridades orçamentárias ^24^.

Nesse contexto, aprofundou-se o subfinanciamento estrutural da área. Sob austeridade fiscal ^9^, a partir de 2018, a União passou a manter a aplicação mínima em saúde congelada em valores reais, desvinculada do crescimento das receitas federais. Essa medida reduziu a capacidade estatal de investir, expandir e qualificar serviços ^10^, afetando particularmente a atenção primária, a vigilância em saúde e o fornecimento de medicamentos essenciais. 

As transferências federais para estados e municípios ficaram aquém das necessidades do SUS, ampliando desigualdades regionais e pressionando os governos municipais e estaduais. Em 2017, a redução dos blocos de transferências do Ministério da Saúde para dois grupos de despesas − custeio e investimento − suscitou questionamentos quanto à preservação da capacidade de coordenação federal, diante dos ganhos de autonomia dos entes subnacionais ^25^. Paralelamente, as emendas parlamentares ganharam relevância na alocação de recursos federais entre os municípios ^26^.

Do ponto de vista político, os ministros da saúde do período expressaram, de diferentes formas, essa inflexão neoliberal, com descontinuidades e ameaças à institucionalidade do SUS. A nomeação de Ricardo Barros como ministro da Saúde, em maio de 2016, em reconhecimento ao apoio de seu partido ao impedimento da presidenta Dilma, simbolizou a convergência entre interesses do setor privado e a condução das políticas públicas. Engenheiro com trajetória parlamentar e apoiado por empresas da saúde, Barros declarou publicamente, no início da gestão, que os direitos constitucionais não seriam sustentáveis diante das restrições orçamentárias e defendeu rever a amplitude do SUS ^27^.

Sua principal iniciativa foi a proposta de “planos de saúde populares”. Para isso, foi formado um grupo de trabalho com representantes do Ministério da Saúde, da Agência Nacional de Saúde Suplementar e da Confederação Nacional das Empresas de Seguros Gerais, da Previdência Privada, da Saúde Suplementar e da Capitalização. A proposta previa planos com cobertura reduzida e baixo custo, voltados à população de baixa renda. Sob o argumento de “ampliar o acesso”, a iniciativa implicava a desregulamentação do setor suplementar e a expansão do mercado de seguros, mas foi rejeitada após análise técnica e participação social ^28^. 

A gestão de Ricardo Barros foi também marcada pela publicação da nova Política Nacional de Atenção Básica, que gerou polêmicas ao flexibilizar a modalidade de organização, a composição de equipes e a carga horária de profissionais ^29^. As políticas de saúde mental sofreram retrocessos por alterações nas regras da rede de atenção psicossocial, que reforçaram as comunidades terapêuticas e os hospitais psiquiátricos ^30^. 

A breve gestão de Gilberto Occhi (abril a dezembro de 2018), último ministro da Saúde de Temer, com formação em gestão pública e carreira na Caixa Econômica Federal, manteve o foco na racionalização administrativa e no controle orçamentário, sem apresentar propostas estruturantes para o fortalecimento do SUS. 

O Conselho Nacional de Saúde (CNS) resistiu às ameaças ao SUS no governo Temer ^31^ por meio do diálogo e do enfrentamento político, inclusive por via judicial. O CNS contestou o ajuste fiscal associado à EC nº 95, de 15 de dezembro de 2016 ^20^, e defendeu o caráter público e universal do SUS, mesmo diante do boicote governamental à sua atuação deliberativa.

Em síntese, o governo Temer promoveu uma reorientação das políticas sociais no Brasil, baseada na contenção fiscal, na redução do papel do Estado e na ampliação dos mecanismos de mercado, prejudicando a trajetória de expansão das políticas de saúde das décadas anteriores. O impacto da EC nº 95/2016 sobre o financiamento federal, somado a reformas regressivas e à desregulamentação de setores estratégicos, fragilizou a capacidade estatal e do SUS na garantia do direito à saúde. Esse conjunto de medidas preparou o terreno para retrocessos maiores no governo subsequente.

## Governo Bolsonaro: descontinuidades, pandemia e negacionismo

Jair Bolsonaro, eleito presidente do Brasil em outubro de 2018, é um político de ultradireita, capitão do Exército na reserva desde 1988 e deputado federal por sete mandatos, que defende interesses de militares e policiais. Ainda que tenha pautas comuns a outros políticos conservadores, um diferencial é a sua postura de defesa da ditadura militar, explicitada em entrevistas à mídia e em sua atuação parlamentar ^4^.

A vitória de Jair Bolsonaro foi favorecida por fatores políticos e institucionais em uma conjuntura crítica. A condução e os desdobramentos da Operação Lava Jato − incluindo a prisão do ex-presidente Luiz Inácio Lula da Silva e o impedimento da presidenta Dilma −, apoiados por atores estatais (Procuradoria Geral da República, Supremo Tribunal Federal, Congresso Nacional), por elites políticas e econômicas e pela mídia, contribuíram para a fragilização da democracia, a inflexão conservadora e a ampliação do espaço para partidos e políticos de extrema direita ^4^ ^,^ ^5^.

Além de fatores políticos, duas alterações nas regras eleitorais influenciaram a eleição de Bolsonaro e de parlamentares de sua base de apoio: a curta duração da campanha eleitoral de 2018 e a redução do prazo para a mudança de filiação partidária antes da eleição ^4^. Também repercutiram na disputa o atentado sofrido por Jair Bolsonaro durante a campanha e a definição de Fernando Haddad como candidato presidencial do Partido dos Trabalhadores (PT) em substituição a Lula, que estava preso e impedido de concorrer. Por fim, a propagação de informações falsas nas redes sociais contribuiu para o crescimento do candidato vitorioso ^4^. 

Jair Bolsonaro venceu as eleições de 2018 no segundo turno com 55,13% dos votos válidos, contra 44,87% de Haddad, tendo sido mais votado entre os homens, enquanto Haddad predominou entre as mulheres. Com exceção do Nordeste, Bolsonaro teve a maioria dos votos em todas as regiões, com expressiva votação nos maiores estados da federação: São Paulo, Minas Gerais e Rio de Janeiro ^4^. 

O governo Bolsonaro deve ser compreendido em duas fases. A primeira corresponde ao ano inicial (2019), marcado por uma agenda de reformas das políticas públicas de orientação neoliberal, conservadora e autoritária. A segunda fase é definida pela resposta à pandemia de COVID-19, que provocou a reorientação de estratégias, ainda que tenham persistido elementos da orientação ideológica, do estilo de governar e da agenda adotada no primeiro momento. 

Destacam-se como características gerais do governo: a retórica antipolítica e o populismo autoritário, expressos na forma de lidar com as instituições e os atores sociais; o discurso crítico ao Estado e à burocracia, associado a medidas de austeridade e restrições de gastos públicos; o ultraliberalismo, como aceno positivo aos mercados; e o protagonismo de lideranças conservadoras e de militares na ocupação de cargos, a fim de agradar à sua base eleitoral ^3^. 

O número de ministérios foi reduzido de 29 para 22, para diminuir despesas e a burocracia. O presidente priorizou critérios de lealdade pessoal e de alinhamento às ideias neoliberais e conservadoras na composição do gabinete. Cabe destacar o economista ultraliberal Paulo Guedes no superministério da Economia e a crescente participação de ministros militares ^32^. Ministros fortemente vinculados à orientação do governo promoveriam retrocessos em políticas de pastas como Ambiente, Educação, Relações Exteriores e no Ministério da Mulher, Família e Direitos Humanos ^3^. Ressalte-se, ainda, a liderança midiática do presidente e a interferência de seus três filhos − um senador, um deputado federal e um vereador − em assuntos do governo. 

A orientação liberal, conservadora e autoritária do governo Bolsonaro se manifestou em reformas institucionais e em políticas públicas. Observou-se baixa valorização da coordenação intergovernamental e do diálogo com comunidades de especialistas de diversas áreas, além de restrições à participação social nas políticas, incluindo constrangimentos à atuação de colegiados de gestão (Quadro 1). 

A política econômica aprofundou mudanças iniciadas no governo Temer, sendo caracterizada por: adoção de medidas fiscais para atender aos limites impostos pela EC nº 95/2016 (reforma da Previdência, contingenciamento e desvinculação de gastos); implantação da reforma trabalhista, que reduziu direitos dos trabalhadores; privatizações de empresas e alterações em estatais. Outras políticas que apresentaram retrocessos foram: a política externa, com deterioração da inserção internacional do Brasil; a ambiental, por fragilização da regulação; a de ciência e tecnologia, por baixos investimentos; a de educação, por restrições orçamentárias, incentivos ao setor privado e conservadorismo; e as políticas relacionadas a gênero, direitos humanos e direitos sexuais e reprodutivos, afetando estratégias voltadas às mulheres, à população LGBTQIA+ e à promoção da igualdade racial ^3^. O termo “desmonte” tem sido utilizado para ressaltar a desconstrução e a magnitude dos retrocessos nas políticas sociais, que incidiram sobre a saúde ^6^.

Na política de saúde, o ano inicial do governo (2019) foi marcado por mudanças polêmicas no Ministério da Saúde e em políticas estratégicas, influenciadas por posições do Governo Federal. O primeiro ministro da saúde do período, Luiz Henrique Mandetta, era deputado federal pelo Estado de Mato Grosso do Sul, por um partido conservador, com experiência como médico militar, gestor municipal e no setor privado em saúde. 

A primeira polêmica se relacionou à interrupção do Programa Mais Médicos, marco do governo Dilma, voltado à provisão de médicos em áreas de vulnerabilidade social, conforme anunciado por Bolsonaro durante a campanha presidencial. Tal programa havia sido criticado por entidades médicas por incorporar profissionais estrangeiros - especialmente cubanos - sem a revalidação nacional dos diplomas. Ao final de 2018, após as eleições, o governo de Cuba suspendeu o convênio com o Brasil, intermediado pela Organização Pan-Americana da Saúde (OPAS), e retirou a maior parte dos médicos cubanos do país. 

Na gestão Mandetta, o governo Bolsonaro lançaria o Programa Médicos pelo Brasil, com objetivos semelhantes, mas exigindo a revalidação nacional dos diplomas obtidos no exterior. Na prática, houve atrasos na implementação do novo programa, o que fez com que ambas as iniciativas convivessem. Outras mudanças nas políticas de APS envolveram alterações nas regras de financiamento − substituição de transferências *per capita* e por equipe por repasses condicionados ao cadastramento das pessoas − e a flexibilização na composição das equipes de Saúde da Família. Em síntese, as mudanças na política de APS expressaram alinhamento às posições de entidades médicas corporativas, com orientação gerencialista e pró-mercado.

A segunda medida polêmica foi a extinção do Departamento de Infecções Sexualmente Transmissíveis, Aids e Hepatites Virais do Ministério da Saúde, que foi substituído pelo Departamento de Doenças de Condições Crônicas e Infecções Sexualmente Transmissíveis. A mudança foi criticada por sanitaristas e pelos movimentos sociais de luta contra a aids, por submeter a coordenação dessa política à estrutura responsável por doenças crônicas. As preocupações relacionaram-se à agenda conservadora do governo, desfavorável às demandas e aos direitos da população LGBTQIA+. 

Nas políticas de saúde mental, foram reiteradas medidas regressivas adotadas no governo anterior, como o reforço às comunidades terapêuticas e aos hospitais psiquiátricos ^30^. Na área de saúde da mulher e direitos sexuais e reprodutivos, destacam-se alterações na rede de atenção materno-infantil e regras que dificultariam o acesso à interrupção da gravidez nos casos previstos em lei (Quadro 1) ^33^. As políticas de controle do tabaco, que requerem coordenação intergovernamental e articulação com a sociedade, foram prejudicadas pela baixa prioridade e pelo enfraquecimento dos mecanismos de gestão colegiada ^34^, com risco de prejuízo ao alcance de suas metas ^35^.

As políticas de saúde no governo Bolsonaro, no entanto, ficariam marcadas pela gestão da pandemia de COVID-19, que atingiu o país a partir de 2020, levando a quase 700 mil óbitos até o final de 2022. Os limites no enfrentamento da crise sanitária extrapolaram o setor da saúde e podem ser imputados, em grande medida, à condução do presidente e seu núcleo de poder, caracterizada pelo autoritarismo e pelo negacionismo. O autoritarismo se expressou na centralização decisória e na imposição de suas ideias, em detrimento do diálogo com outros governos, autoridades sanitárias, científicas e grupos sociais. Já o negacionismo se manifestou na desconsideração da gravidade da emergência sanitária, na rejeição de evidências científicas para seu enfrentamento e na recomendação de medidas sem base técnico-científica. 

O Ministério da Saúde foi marcado por instabilidade e fragilização institucional, com quatro ministros no período. Limitações na governança nacional da resposta à pandemia se expressaram na baixa valorização das instâncias do SUS de coordenação intergovernamental (comissões intergestores) e de participação social (conselhos de saúde) e no escasso diálogo do governo com especialistas e diferentes grupos sociais. 

Em que pesem essas características gerais, identificam-se diferentes momentos no enfrentamento da pandemia de COVID-19 no Brasil (Quadro 2). 

**Quadro 2:** Momentos do enfrentamento da pandemia de COVID-19 no Brasil, 2020-2022.

TENTATIVAS DE COORDENAÇÃO PELO MINISTÉRIO DA SAÚDE (JAN-ABR/2020)	POLARIZAÇÃO POLÍTICA “SAÚDE X ECONOMIA” (ABR-DEZ/2020)	VACINAÇÃO LENTA, ALTA MORTALIDADE, INVESTIGAÇÃO PARLAMENTAR (JAN-JUN/2021)	EFETIVIDADE DA VACINAÇÃO X NEGACIONISMO (JUN/2021-DEZ/2022)
Limites das ações federais no fechamento de fronteiras, medidas econômicas e sociais Medidas de distanciamento físico dependentes de estados e municípios Preparação inicial: nova legislação, instalação do Centro de Operações de Emergências (COE) nacional, treinamento de laboratórios nacionais e de outros países da América Latina para diagnóstico. Esforços de coordenação e informes diários do Ministério da Saúde Ênfase: atendimento hospitalar Dificuldades na importação de suprimentos e equipamentos Conflitos entre posições do Presidente e do Ministério da Saúde	Conflitos: presidente, autoridades de saúde e especialistas Mudanças no Ministério da Saúde: duas trocas de ministro, alterações na condução Decisões do Governo Federal não baseadas em evidências científicas Resistência às medidas de distanciamento físico, pressões para normalizar a economia Vacinas: acordos de transferência tecnológica X conflitos sobre compras e efetividade Instabilidade dos sistemas nacionais de informação Iniciativas subnacionais (p. ex.: Consórcio Nordeste, envolvendo governadores, autoridades de saúde, parceiros), de organizações públicas (Fiocruz, universidades), de veículos de imprensa e de movimentos sociais para prover informações confiáveis sobre a COVID-19 e promover outras medidas de controle	Produção doméstica de vacinas (Fundação Oswaldo Cruz [Fiocruz] e Butantan): dependente de importações de ingrediente farmacêutico ativo (IFA) Vacinação gradual pelo Sistema Único de Saúde (SUS), de acordo com disponibilidade das vacinas. Março de 2021: mudanças no Ministério da Saúde Instalação de Comissão Parlamentar de Inquérito (CPI) sobre a resposta do Governo Federal (CPI da Pandemia) Conflitos entre autoridades governamentais e especialistas/cientistas	Alcance de alta cobertura vacinal em adultos com o esquema básico protege contra hospitalizações e mortes Ciclo completo de produção nacional de vacinas pela Fiocruz Aumento da disponibilidade de vacinas e testes Apesar das evidências científicas sobre a efetividade vacinal, notícias falsas e campanhas antivacina geram hesitação vacinal e baixa cobertura entre as crianças e menor cobertura de doses de reforço em adultos (após o esquema básico) Relatório final da CPI da Pandemia aponta omissões e crimes de autoridades a serem apurados

Fonte: elaboração própria.

O momento inicial, de janeiro a abril de 2020, caracterizou-se por tentativas do Ministério da Saúde de coordenar uma resposta à COVID-19, diante dos primeiros casos da doença na China e de sua rápida disseminação na Europa. A Organização Mundial da Saúde (OMS) declarou Emergência de Saúde Pública de Importância Internacional em 30 de janeiro; já o Brasil declarou emergência nacional em 3 de fevereiro, mesmo sem casos registrados, e instituiu o Centro de Operações de Emergências em Saúde Pública. Ainda em fevereiro, o Ministério da Saúde aprovou uma lei com regras para isolamento, quarentena, notificação compulsória e outras medidas de controle. Por meio da Fundação Oswaldo Cruz (Fiocruz), instituição federal de ciência e tecnologia em saúde, iniciou-se a produção nacional de testes diagnósticos e realizou-se o treinamento de laboratórios de nove países da América Latina para o processamento de testes de COVID-19. O Ministério da Saúde realizava transmissões diárias na televisão, com informações sobre a doença no mundo e no país.

Esse contexto foi marcado pela prontidão na resposta do Ministério da Saúde, favorecida pela existência do SUS, pela tradição do país na vigilância em saúde e pela experiência prévia na resposta a emergências sanitárias ^36^. No âmbito dos serviços, enfatizou-se a atenção hospitalar, com orientações, expansão de leitos e organização de hospitais de campanha temporários em vários estados, porém com pouca valorização da atenção primária na resposta.

As recomendações do Ministério da Saúde, baseadas em critérios técnicos e nas evidências científicas disponíveis, colidiram com as posições do presidente Bolsonaro, contrárias ao distanciamento físico. As medidas federais de fechamento de fronteiras, restrição à mobilidade e promoção do distanciamento físico foram tímidas, dependendo dos governos estaduais e municipais. Os conflitos culminaram na demissão do ministro Mandetta em abril de 2020, seguida da saída de outros dirigentes.

O segundo momento, de abril a dezembro de 2020, foi marcado pela exacerbação da polarização “saúde *versus* economia”. A recomendação técnica de manter medidas de distanciamento físico, devido à ausência de vacinas, esbarrou na resistência de agentes econômicos e de governantes. As medidas nacionais de proteção social, ao emprego e às empresas, foram tardias e insuficientes, com deslocamento da responsabilidade para estados e municípios. Exacerbaram-se os conflitos entre o presidente, pesquisadores e especialistas em saúde. O Ministério da Saúde passou por momentos de instabilidade, com duas trocas de ministro. O sucessor de Mandetta, Nelson Teich, médico oncologista, se demitiu após menos de um mês no cargo (em maio de 2020). 

Designou-se, então, um novo Ministro da Saúde, o general Pazuello, sem formação específica ou experiência na gestão do SUS, visando melhorar a logística da resposta à crise. A presença de militares em cargos civis na saúde expandiu-se em 74% entre 2018 e 2020, acompanhando a militarização da gestão federal no período. Ao final de 2020, havia 10 ministros militares, quase a metade do total ^32^. 

Para evitar conflitos com o presidente, o Ministério da Saúde não enfatizava medidas recomendadas por especialistas, como o distanciamento físico e o uso de máscaras. Ademais, foi omisso ou endossou posições sem base científica, inclusive a utilização de medicamentos sem eficácia comprovada contra a COVID-19. Houve episódios de instabilidade na provisão de dados relativos à doença, com a adoção de alternativas por estados, instituições científicas e a mídia para consolidar tais informações. Outra polêmica foi a relutância do Governo Federal em firmar acordos de compra com empresas produtoras de vacinas contra COVID-19, que se encontravam ainda em desenvolvimento.

Por outro lado, avançaram nesse período negociações relativas à transferência tecnológica para produção nacional de vacinas. A Fiocruz, instituição federal, liderou um desses processos, com anuência do Ministério da Saúde, para produzir a vacina com a tecnologia AstraZeneca-Oxford (Reino Unido). Já o Instituto Butantan, do Estado de São Paulo, firmou um acordo com a empresa Sinovac (China) para produzir a vacina CoronaVac. 

O terceiro momento, de janeiro a junho de 2021, foi caracterizado pelo início da vacinação no país, com as vacinas produzidas nacionalmente pela Fiocruz e pelo Instituto Butantan. O ritmo de disponibilização das vacinas foi prejudicado pela dependência da importação dos Ingredientes Farmacêuticos Ativos (IFA), que sofreu atrasos devido à sua insuficiência no mercado para atender à demanda global. Ressalte-se a existência de um robusto Programa Nacional de Imunizações (PNI) no país e o predomínio na população brasileira de uma visão positiva sobre as vacinas, como intervenção efetiva para prevenir doenças e como direito social. Ainda assim, as vacinas foram objeto de conflitos federativos ^37^ e do negacionismo do presidente Bolsonaro quanto à sua eficácia, o que incentivou a disseminação de notícias falsas, provocando desinformação e hesitação vacinal. Em março de 2021, o ministro Eduardo Pazuello foi substituído por Marcelo Queiroga, médico cardiologista, o quarto ministro da Saúde no período da pandemia, que permaneceria até o final do governo Bolsonaro.

Os problemas na resposta federal à pandemia fizeram com que o Brasil apresentasse alarmantes taxas de mortalidade por COVID-19 e excesso de mortalidade geral. Em consequência, o Senado instalou uma Comissão Parlamentar de Inquérito − a CPI da Pandemia − que analisou uma documentação extensa e arguiu autoridades e especialistas.

No quarto momento, de junho de 2021 a dezembro de 2022, alcançou-se alta cobertura vacinal contra COVID-19 no país, especialmente entre adultos, com evidências científicas sobre a efetividade das vacinas na redução de hospitalizações e mortalidade pela doença ^38^. A Fiocruz passou a produzir o IFA da vacina AstraZeneca-Oxford e aumentou o fornecimento de testes. O Brasil também começou a comprar vacinas de produtores internacionais, especialmente as de tecnologia de mRNA, e os autotestes rápidos chegaram às farmácias. Em que pesem as evidências sobre a efetividade das vacinas e a inefetividade de determinados medicamentos no manejo da COVID-19, notícias falsas se disseminam, fomentando a hesitação vacinal, especialmente quanto à imunização de crianças e às doses de reforço. 

Em outubro de 2021, o Senado divulgou o relatório final da CPI da Pandemia (Quadro 1), recomendando o indiciamento de mais de 60 pessoas, entre as quais o presidente Bolsonaro, ministros − incluindo dois da Saúde, Pazuello e Queiroga −, dirigentes do Ministério da Saúde, três filhos do presidente, assessores da Presidência, parlamentares, médicos, blogueiros, empresários, entre outros. O presidente foi indiciado por dez crimes, incluindo crimes de responsabilidade e contra a humanidade. O relatório trouxe propostas relativas à criminalização de notícias falsas, gestão do SUS, resposta a calamidades, responsabilização de agentes públicos e apoio e reparação às vítimas da COVID-19. O Procurador-Geral da República, no entanto, arquivou o documento.

Ao final de 2022, com o encerramento do governo Bolsonaro, o país registrava quase 700 mil mortes por COVID-19, muitas das quais poderiam ter sido evitadas. Houve expressivo excesso de mortalidade, possivelmente associado também a outras causas. Além da morbidade e mortalidade pela doença, assinale-se a piora de outros indicadores de saúde − em parte porque as pessoas evitaram ou tiveram dificuldades em acessar serviços de saúde durante os momentos críticos da pandemia − e o aumento da hesitação vacinal no país. As repercussões econômicas e sociais foram drásticas, atingindo milhões de famílias.

O Quadro 3 resume as características dos governos Temer e Bolsonaro, considerando elementos do contexto, do processo político e do conteúdo das políticas de saúde.

**Quadro 3:** Contexto, processo político e conteúdo da política nacional de saúde de 2016 a 2022, segundo os governos.

DIMENSÃO	GOVERNO TEMER (05/2016-12/2018)	GOVERNO BOLSONARO (01/2019-12/2022)
Contexto	- Processo de impedimento de Dilma Rousseff: o vice-presidente Temer assume em maio de 2016 em caráter interino e, em agosto, de forma efetiva, até o final de 2018 - Políticas econômicas neoliberais, com ênfase na austeridade e redução de gastos, orientadas pela *Uma Ponte para o Futuro* ^15^ - Configuração dos ministérios e perfil dos ministros: fusão ou extinção de pastas, predomínio de homens brancos de perfil conservador - *Emenda Constitucional nº 95*/2016 - teto de gastos - Propostas de reforma trabalhista e previdenciária, com redução de direitos dos trabalhadores e sociais - Prisão de Lula, que é impedido de concorrer nas eleições de 2018 - Campanha de 2018: mudanças nas regras eleitorais, atentado a Bolsonaro - Eleições: vitória de Bolsonaro sobre Fernando Haddad (no 2º turno)	- Reforma ministerial: redução do número de ministérios por extinção ou fusão de pastas; criação do superministério da economia (comandado por Paulo Guedes) - Ministros alinhados com agenda neoliberal e conservadora e militares aumentam em áreas estratégicas - Militarização na ocupação de cargos civis na administração federal - Políticas econômicas ultraliberais e medidas para favorecer os mercados - Conservadorismo leva a retrocessos em políticas pró-diversidade e equidade - Retrocessos nas políticas externa, ambiental, de educação, entre outras - Pandemia de COVID-19 atinge duramente o país entre 2020 e 2022: resposta federal marcada por insuficiente coordenação de políticas, negacionismo, autoritarismo e contradições - Instalação da Comissão Parlamentar de Inquérito (CPI) da Pandemia em 2021
Processo político na saúde	Gestões ministeriais Ricardo Barros (Mai/2016-Abr/2018): - Engenheiro civil e empresário. - Deputado Federal − Partido Progressista (PP − Paraná); ex-prefeito de Maringá (Paraná) - Questionamento sobre a amplitude dos direitos sociais e do SUS - Composição conservadora no secretariado do Ministério da Saúde Gilberto Occhi (Abr/2018-Dez/2018): - Advogado e servidor de carreira da Caixa Econômica Federal - Filiado ao PP; ex-ministro de Cidades e de Integração Nacional no governo Dilma (entre 2014-2016) - Ênfase na gestão orçamentária e racionalização administrativa Relações com outros atores: - Sinergia com orientações de autoridades econômicas - Privilegiamento de atores privados - Resistência do Conselho Nacional de Saúde e de outros atores setoriais	Gestões ministeriais Luiz Henrique Mandetta (Jan/2019-Abr/2020): - Médico ortopedista e proprietário rural - Deputado Federal - Democratas (DEM - Mato Grosso do Sul) - Experiência na gestão pública e privada em saúde - Composição mista no ministério, com perfil conservador e quadros técnicos - Posições técnicas no início de enfrentamento da COVID-19, gerando conflitos com o presidente Bolsonaro e queda do ministro Nelson Teich (Abr/2020-Mai/2020): - Médico oncologista e empresário. - Pediu demissão após menos de um mês no cargo. Eduardo Pazuello (Mai/2020-Mar/2021): - General do Exército, sem formação na saúde. - Nomeado como ministro interino em maio e efetivo em setembro de 2020 - Crescente ocupação de cargos do Ministério da Saúde por militares - Alinhamento público às orientações do presidente no enfrentamento da pandemia, mas autorização para produção nacional de vacina pela Fundação Oswaldo Cruz (Fiocruz) Marcelo Queiroga (Mar/2021-Dez/2022): - Médico cardiologista, com experiência de gestão no setor privado - Alinhamento com o presidente - Continuidade em relação à gestão anterior Relações com outros atores: - Apoio de entidades médicas nacionais de perfil conservador - Influências do negacionismo do Presidente e de outros atores - Fragilização técnica e crescente militarização no Ministério da Saúde, com instabilidade na gestão da pandemia - Restrições à gestão colegiada e à participação social - Conflitos com especialistas e outras esferas de governo. - Governos estaduais e municipais adotam políticas variadas - Resistências de instituições científicas, órgãos de saúde (inclusive no interior do Ministério da Saúde, Fiocruz, Agência Nacional de Vigilância Sanitária), de profissionais de saúde, de movimentos sociais
Conteúdo da política de saúde	- Contenção de gastos por restrições orçamentárias (efeitos da *Emenda Constitucional nº 95*) - Proposta de planos populares de saúde para população de baixa renda: conformação de grupo de trabalho, porém resistências e rejeição - Mudanças nas regras de transferências federais, que passam a ser em dois blocos: custeio e investimentos - Mudanças na Política Nacional de Atenção Básica (2017) com flexibilização na composição de equipes e no número de Agentes Comunitários de Saúde por equipe - Mudanças nas políticas de Saúde Mental e Drogas reforçam os hospitais e comunidades terapêuticas, criticadas por especialistas por colidirem com as orientações da reforma psiquiátrica brasileira e os avanços do modelo de atenção no Sistema Único de Saúde	- Anúncio da interrupção do Programa Mais Médicos (marco do governo anterior) e lançamento do programa Médicos pelo Brasil, com exigência de revalidação de diplomas de médicos formados no exterior. Na prática, convivem no país no período profissionais contratados por ambos os programas - Mudanças nas regras de financiamento da atenção básica, com relativa fragilização da Estratégia Saúde da Família - Políticas específicas afetadas pela agenda conservadora do governo (ex: saúde da mulher, população LGBTQIA+), por restrições financeiras e por redução da participação social - Mudanças nas regras relativas à rede de atenção materno-infantil e de autorização para a realização da interrupção da gravidez nos casos previstos em Lei. - Continuidade nas mudanças nas políticas de Saúde Mental e Drogas adotadas no governo anterior, criticadas por especialistas por colidirem com as orientações da reforma psiquiátrica brasileira e os avanços do modelo de atenção no Sistema Único de Saúde (SUS) - Gestão da resposta à pandemia de 2020: diferentes momentos (ver Quadro 2) e contradições; tensões entre estratégias técnicas (ex: decisão de produção nacional de vacinas) x medidas sem base científica

Fonte: elaboração própria, com base em diversas fontes bibliográficas e documentais.

## Discussão

O período de 2016 a 2022, correspondente aos governos Temer e Bolsonaro, representou uma conjuntura crítica para as políticas de saúde no Brasil, devido a acontecimentos em dois planos principais. 

O primeiro plano é o político-institucional. A crise política e o impedimento de Dilma Rousseff − identificado por muitos como um golpe ^39^ ^,^ ^40^ −, alçaram o Vice-presidente Temer ao poder e favoreceram a adoção de reformas neoliberais, pautadas pela austeridade, pelo privilegiamento de interesses privados e por uma visão gerencialista das políticas. O governo Bolsonaro deu continuidade a essas reformas, associadas a uma pauta conservadora e a mecanismos antidemocráticos, como proibições legais a instâncias colegiadas, crescente ocupação de cargos estratégicos por pessoas sem qualificação técnica − incluindo militares ^32^ −, desrespeito às instituições e descontinuidades em políticas públicas, por constrangimentos econômicos, ideias neoliberais ou conservadorismo. Ou seja, as agendas e medidas dos governos Temer e Bolsonaro colidiram com os princípios e condições necessários à consolidação do SUS.

O segundo plano é o sanitário, dada a magnitude da pandemia de COVID-19 e suas trágicas repercussões no Brasil. A emergência sanitária alcançou o país em um cenário político-institucional adverso, quando os efeitos negativos da agenda neoliberal e conservadora já afetavam as políticas públicas e o SUS. Ainda que fatores estruturais, institucionais e societais tenham influenciado a resposta do Brasil à pandemia ^11^, os elementos político-conjunturais, com destaque para as posições do presidente Bolsonaro, foram decisivos para explicar os péssimos resultados observados, em um país que conta com um sistema universal de saúde e com instituições científicas sólidas. O relatório da CPI da Pandemia apontou evidências de omissões e crimes cometidos por governantes que mereceriam apreciação pelo Judiciário.

Em uma conjuntura crítica, nos planos político-institucional e sanitário, as políticas de saúde foram atingidas por: contingenciamento e instabilidade orçamentária; fragilização dos órgãos federais; ocupação de postos estratégicos por pessoas sem qualificação ou com posicionamentos contrários aos princípios do SUS; desrespeito aos mecanismos de participação social; descontinuidades em políticas (como as de atenção básica, saúde mental, saúde da mulher e controle do tabaco); negacionismo de governantes e decisões sem base científica. Portanto, a inflexão política a partir de 2016 teve repercussões na saúde no período analisado, incluindo a fragilização institucional, o crescimento do negacionismo científico, retrocesso em políticas específicas, piora de indicadores sanitários e mortes potencialmente evitáveis no contexto da pandemia. 

Por outro lado, nessa conjuntura crítica, houve resistências ao desmonte de instituições e políticas no interior do Estado e na sociedade. Servidores públicos, pesquisadores, gestores, profissionais de saúde, sociedades de especialistas, colegiados participativos, movimentos sociais e alguns políticos se mobilizaram para defender o SUS e a saúde da população. 

Ainda que o período tenha representado uma conjuntura crítica para a saúde, cabe indagar se tais eventos produziram mudanças institucionais substantivas que alterassem os rumos do sistema de saúde brasileiro. Essa dúvida surge porque, apesar do debate estar associado à expectativa de mudança institucional, a relação entre ambas não é inequívoca: mudanças institucionais podem ocorrer de forma gradual e incremental, e não apenas no contexto de conjunturas críticas ^13^, e conjunturas críticas nem sempre levam a transformações institucionais ^14^.

A conjuntura analisada poderia ter provocado profundas transformações institucionais na saúde, em diferentes sentidos. O estudo evidenciou a complexidade desse contexto e disputas entre diferentes projetos para o Brasil e o sistema de saúde. Por um lado, o neoliberalismo, o conservadorismo e o autoritarismo fragilizaram as instituições democráticas, as estruturas estatais e as políticas públicas, mas não conseguiram destruí-las durante o período analisado. Exemplos de transformações radicais contrárias ao SUS seriam: mudanças constitucionais que alterassem o direito à saúde e as regras do sistema; o desmantelamento de órgãos e entidades estatais; a destruição de capacidades estatais em áreas estratégicas (regulação, vigilância em saúde, prestação de serviços, produção de insumos); o desmonte das instâncias colegiadas do SUS, de coordenação federativa e de participação social; e a substituição de um modelo universal por outro ancorado na expansão de planos e seguros privados. 

Por outro lado, o início da emergência sanitária suscitou expectativas quanto à inflexão na atuação dos Estados nacionais, no sentido de maior responsabilidade na proteção social e em saúde ^7^ ^,^ ^8^. No Brasil, a conjuntura política era desfavorável à expansão estatal, mas a pandemia pode ter freado reformas neoliberais radicais ao evidenciar a importância das instituições públicas na resposta imediata à crise, em especial as científicas, as de proteção social e o SUS. Atores defensores do SUS formaram coalizões com sociedades de especialistas, movimentos sociais e parlamentares de orientações políticas variadas. A título de exemplo, o Congresso Nacional autorizou aportes orçamentários extraordinários para o enfrentamento da emergência. A capilaridade nacional do SUS e a densidade técnico-política de suas instituições e serviços, sustentadas por atores e interesses diversos, foram fundamentais para a resposta, ainda que esta tenha sido prejudicada por posições da Presidência contrárias às recomendações científicas e pela atuação insuficiente do Governo Federal.

Em síntese, a conjuntura crítica teve efeitos imediatos nas organizações e nas políticas de saúde no período analisado. Porém, o estudo não permitiu identificar transformações estruturais e institucionais radicais, no sentido da expansão ou retração da atuação do Estado na saúde, de mudanças nas regras do SUS ou da destruição ou fortalecimento de suas organizações. Em que pesem as pressões e os constrangimentos, nem as instituições de saúde, nem as democráticas chegaram a ser destruídas nesse período. Em outra perspectiva, as expectativas de expansão do Estado e de fortalecimento das instituições públicas, em decorrência da experiência da crise, não se concretizaram. 

Alguns fatores podem explicar por que não se observaram, na conjuntura analisada, transformações radicais nas instituições de saúde no Brasil. O primeiro, de caráter histórico-estrutural, seria a configuração singular da saúde no país, em que um sistema de saúde universal, criado há mais de três décadas, convive com um setor privado vigoroso, acomodando interesses diversos. O segundo seria a densidade institucional do SUS, que se manifesta em diferentes dimensões: na jurídica, pela proteção constitucional ao direito à saúde e aos princípios do SUS; na organizacional, pela capilaridade territorial da rede de serviços; na federativa, pelas competências comuns, interdependência e mecanismos de coordenação entre esferas de governo; na técnica, pelo conhecimento e experiência acumulados nos órgãos e serviços públicos; na sociopolítica, pela diversidade de atores envolvidos com o SUS, incluindo gestores, profissionais, conselhos participativos, comunidades epistêmicas e movimentos sociais.

Esses dois fatores − a configuração histórico-estrutural da saúde e a institucionalidade do SUS − favoreceriam certa “dependência de trajetória” ^13^ o que explicaria a relativa estabilidade institucional e sua sustentação por coalizões de atores que resistem a alterações nas regras formais. 

O terceiro se relaciona à concomitância de agendas e forças de sentidos opostos na conjuntura crítica analisada. De um lado, a agenda neoliberal, conservadora e antidemocrática levou a retrocessos nas políticas e provocou resistências. De outro lado, a gravidade da emergência sanitária, ao exigir a intervenção estatal imediata, pode ter freado o ritmo das reformas neoliberais. Porém, a expectativa de fortalecimento do Estado e do SUS em decorrência da crise não se concretizou, diante do cenário político desfavorável. 

O quarto fator concerne ao recorte temporal do estudo, considerando o curto e recente período analisado, não tendo sido possível, até o momento, identificar efeitos de maior duração ou tardios. No que diz respeito à dimensão política, nas eleições presidenciais de 2022, pela primeira vez desde a democratização, um presidente em exercício − Jair Bolsonaro − não foi reeleito para um segundo mandato, o que interrompeu a implementação de sua agenda de governo. A tentativa de golpe de Estado após a vitória eleitoral de Lula foi frustrada, desdobrando-se no julgamento e condenação de diversos acusados. Em que pesem as ameaças, as instituições democráticas brasileiras mostraram certa resistência. No terceiro mandato de Lula, iniciado em 2023, foram revertidas medidas adotadas nos governos Temer e Bolsonaro na saúde, o que requer estudos futuros. 

Nesse cenário, o estudo contribui para o debate teórico sobre a dinâmica das políticas públicas, ao evidenciar que conjunturas críticas podem produzir efeitos políticos e sanitários relevantes, sem necessariamente resultar em mudanças estruturais ou institucionais radicais, especialmente em contextos marcados por alta densidade institucional e disputas entre projetos antagônicos. Assinala-se, ainda, que as repercussões da conjuntura crítica dependem da sua duração e da interação entre agentes e coalizões políticas. 

Ressaltam-se como limitações do estudo o caráter recente dos acontecimentos e a não realização de entrevistas com atores envolvidos nas políticas, parcialmente contornada pela diversidade de fontes documentais e pela consulta a declarações de governantes, gestores e especialistas disponíveis em páginas oficiais, matérias jornalísticas e vídeos. Apesar de menções à composição dos governos, que expressam sua orientação conservadora ou autoritária − por exemplo, o ministério formado por homens brancos no Governo Temer e a presença dos militares no Governo Bolsonaro −, este estudo não aprofundou a análise da influência do perfil dos atores e das relações de poder na direcionalidade das políticas de saúde. Cabe realizar pesquisas que explorem como a composição e a diversidade no governo, em diferentes dimensões (partidária, regional, de gênero, étnico-racial e de base de apoio), repercutem nas políticas setoriais. Outro limite diz respeito ao foco na política nacional de saúde, com ênfase no contexto e na identificação de mudanças que colidiram com os princípios do SUS ou conquistas prévias, denunciadas na comunidade de especialistas e na mídia. Assim, o estudo não se aprofundou em políticas específicas nem explorou fatores internacionais e subnacionais que influenciam as políticas de saúde.

Na perspectiva da saúde global, após a OMS decretar o fim da Emergência de Saúde Pública de Importância Internacional em 2023, a COVID-19 passou a ser acompanhada como mais uma doença infecciosa em vários países, inclusive no Brasil. Porém, a crise gerou desdobramentos relevantes, como a aprovação em 2025 do Acordo sobre Pandemias ^41^, cuja implementação precisa ser acompanhada.

Em um cenário de transformações globais que impactam a saúde, é importante desenvolver estudos que explorem as repercussões dos acontecimentos da última década, inclusive da pandemia, a médio e longo prazo, considerando fatores globais, regionais e nacionais. Além disso, diante de projetos conflitantes para o país, é fundamental analisar continuidades e mudanças nas políticas de saúde em diferentes contextos, incluindo a qualidade da democracia e a orientação política dos governos. Nessa agenda de pesquisa, cabe considerar que mudanças graduais e incrementais podem ter efeitos relevantes nas políticas públicas ^13^ e nos rumos do sistema de saúde brasileiro. 

Por fim, além da preparação para futuras emergências, a consolidação do SUS depende do enfrentamento de problemas estruturais, como o financiamento insuficiente, a alta dependência de importações de insumos e o caráter das relações Estado-mercados em saúde. Além disso, requer ainda mudanças no padrão de desenvolvimento econômico-social e a afirmação dos valores democráticos, aspectos fundamentais para a construção de uma sociedade menos desigual.

## Data Availability

As fontes de informação utilizadas no estudo estão indicadas no corpo do artigo.
